# Anisotropic Roughening of a Au(111) Single-Crystal Electrode Surface in HClO_4_ Solution during Oxidation–Reduction Cycles

**DOI:** 10.1021/acs.jpcc.5c01177

**Published:** 2025-05-05

**Authors:** Saeid Behjati, Mojtaba Hajilo, Maximilian Albers, Marc T. M. Koper

**Affiliations:** † Leiden Institute of Chemistry, Leiden University, P.O. Box 9502, Leiden 2300 RA, The Netherlands; ‡ Chemistry Department, Sharif University of Technology, P.O. Box 11155-3615, Tehran 19166, Iran

## Abstract

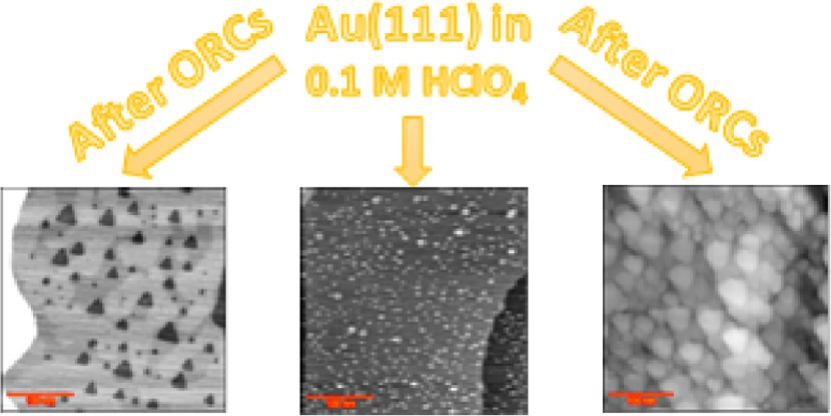

This study investigates the inhomogeneous roughening of a Au(111) single-crystal electrode surface during oxidation–reduction cycles (ORCs) in a 0.1 M perchloric acid (HClO_4_) solution using electrochemical scanning tunneling microscopy (EC-STM). The results reveal that, even in ultrapure HClO_4_, the presence of minor impurities can lead to three distinguishable surface evolutions, on one and the same crystal: surface roughening by the formation of adatom and vacancy islands, gold dissolution resulting in vacancy island formation (in conjunction with step-line recession), and the surface remaining intact even during oxidation–reduction cycling. The impact of trace impurities, specifically sulfate (SO_4_^2–^) and chloride (Cl^–^), on the surface structure development is investigated by adding 10 μM of H_2_SO_4_ and HCl to the HClO_4_ solution. Our results reveal that sulfate significantly promotes uniform roughening, while chloride accelerates gold dissolution and step-line recession. These findings highlight the critical role of minor impurities in altering the electrochemical behavior of gold surfaces and how sensitive the local evolution of the surface structure is to these effects.

## Introduction

Gold and its alloys are among the most important materials for various electrochemical applications. Known for its high chemical inertness compared to metals like platinum and its stability in aqueous electrolytes, gold frequently acts as an inert electrode in diverse electrochemical reactions.^1,2^ For example, gold is utilized in electroplating,^3,4^ in electrochemical sensors,^5^ and as electrocatalysts for various reactions, specifically for CO_2_ reduction, selective oxidation reactions, and oxygen reduction reaction (ORR) to hydrogen peroxide.^6−8^ Additionally, it plays a role in energy storage technologies such as supercapacitors and batteries.^9,10^ Moreover, gold is employed in electroanalytical methods for the precise quantitative analysis of analytes in solution.^11^ In particular, gold constitutes an important model system in the context of fundamental principles in electrochemistry, as well as the mechanisms and kinetics of the initial stages of metal oxidation and reduction.^12−20^ Despite its high chemical inertness being advantageous for these applications, the extensive use of gold-based devices is limited by reactivity loss and reduced durability due to structural degradation and dissolution of gold during prolonged use. In fact, oxidation–reduction cycles (ORCs) etch gold when sufficient positive potentials are applied.^21−23^ Since elucidating the dissolution mechanism of noble metals like gold is important for both industrial applications and fundamental science, the structure and characteristics of polycrystalline and single-crystal surfaces of gold in acidic media, especially in the presence of anions like perchlorate,^23−28^ sulfate,^21,24,29^ and chloride,^23,28,30−32^ have been extensively studied by electrochemical and surface characterization techniques.

At sufficiently positive potentials, sulfate or chloride anions specifically adsorb on the gold surface, forming an ordered sulfate^21,33,34^ or chloride adlayer.^35,36^ In contrast, perchlorate is considered as a weakly adsorbed anion.^23,31,37^ As a result, even trace amounts of chloride or sulfate impurities in HClO_4_, which strongly adsorb and may alter the nature of the double layer in a perchlorate solution, can significantly affect the electrochemical behavior of gold, in terms of both voltammetry and surface structure. In situ electrochemical scanning tunneling microscopy (EC-STM) is an effective technique for capturing the atomic-scale surface evolution of Au(111) from its pristine state through various stages of electrochemical treatment.^24^

Studies of the Au(111) electrode in a perchloric acid electrolyte after oxidation–reduction cycles (ORCs) with EC-STM have yielded widely varying results. For instance, one study reported the formation of adatom and vacancy islands after ORCs,^30^ while another observed worm-like islands.^26^ Contradictorily, other studies noted the development of pits post-ORCs,^27,28,31,32^ with the shape and roughness of these pits varying significantly across different reports.^24^ Additionally, some studies identified areas with diverse structures, including variations in pit size,^24,27^ regions with differing heights and roughness,^26,27^ and unexpectedly, areas with less pits and adatom islands adjacent to the initial scan area.^27^ These diverse findings underscore the complexity of the double-layer structure in HClO_4_ and highlight the necessity for further investigations in this area of electrochemistry.

In this paper, we conduct a comprehensive study and analysis of the oxidation–reduction process of Au(111) in perchloric acid to gain a deeper understanding of the roughening approach in a perchlorate solution. Our aim is to identify the primary behavior of the gold surface amidst the various possible behaviors influenced by impurities. Specifically, we investigated how chloride and sulfate impurities in the perchlorate solution affect the double-layer structure and gold surface dynamics. Our findings reveal that the inconsistencies observed in a pure perchlorate solution are very likely attributable to the presence of these minor impurities.

## Experimental Section

### EC-STM Measurements

Electrochemical scanning tunneling microscopy (EC-STM) images were obtained using a custom-built instrument developed at the Leiden Institute of Chemistry (LIC) at Leiden University. The EC-STM cell is constructed from PEEK. Detailed descriptions of the instrument’s design and construction are provided in the Supporting Information of our recent paper.^38^ Tips were crafted from a platinum/iridium wire (90/10) by using the pulling–cutting method. To minimize extra faradaic current, a layer of hot melt adhesive (EVA-copolymer, synthetic resin, wax, and stabilizer, brand: C.K.) was applied, leaving the apex exposed. The working electrode (WE) was a disk-shaped single-crystal Au(111) electrode (10 mm diameter) with a gold wire welded at the back. This crystal, cut with 0.1° accuracy and polished to a 30 nm roughness by the Surface Preparation Laboratory (SPL) in The Netherlands, was annealed with a butane flame torch to an orange color for five minutes and then cooled in air above ultrapure water to prevent contamination. A high-purity gold wire served as the counter electrode (CE), and a reversible hydrogen electrode (RHE, Hydroflex, and Gaskatel) was used as the reference electrode (RE). The distance between the WE and RE was approximately 7 mm, to minimize the ohmic drop during the voltage sweep. Images were recorded in constant tunneling current mode with a current set point ranging from 50 to 150 pA. The tip bias ranges from 10 to 50 mV. To maximize the distance between the tip and the sample during CV application, the current set point was set to zero. The electrochemical voltage was then adjusted to the “rest potential”, allowing the tip to approach as the current set point increased, resulting in the appearance of tunneling current. Throughout the experiment, the EC-STM chamber was purged with ultrahigh-purity argon gas to reduce the risk of oxygen or other gas dissolution into the cell. All of the experiments start with applying an electrochemical potential of 0 V vs RHE, increasing the potential to the point of the lifting of the reconstruction, followed by applying the ORCs. In the course of the experiments, the lower potential windows varied from 0.9 to 0.8 V to rule out the possibility of not reducing the oxidized surface and its effect on the observed results. Above 0.8 V, Au(111) should not reconstruct.^37^

### Electrochemical Cell and Electrolyte

For standard electrochemical experiments, a custom-made Pyrex glass cell was employed. All glassware and plastic components were cleaned by soaking in a permanganate solution (0.5 M sulfuric acid and 1 g/L potassium permanganate) for at least 12 h before each experiment. After thorough rinsing with Milli-Q water, the components were treated with a diluted piranha solution (3:1 mixture of sulfuric acid (H_2_SO_4_) and hydrogen peroxide (H_2_O_2_), diluted with water) to remove any manganese oxide and permanganate residues. To ensure that all traces of the diluted piranha solution were eliminated, the parts were boiled at least five times. The electrolyte was prepared using ultrahigh-purity (UHP) Milli-Q water (resistivity > 18.2 MΩ cm) and included HClO_4_ (70% Suprapur Sigma-Aldrich), H_2_SO_4_ (96% Suprapur Sigma-Aldrich), HCl (30% Suprapur Sigma-Aldrich), and ROTIPURAN Ultra/Supra HClO_4_. The solution was degassed with ultrahigh-purity argon gas for at least 30 min before use. All measurements were conducted at room temperature (293 K).

## Results and Discussion

Many EC-STM experiments were conducted to investigate the morphological changes of a well-annealed Au(111) surface during oxidation–reduction cycles (ORCs) in a 0.1 M HClO_4_ solution. Three main surface responses were observed: roughening and nanoisland formation (adatom and vacancy islands), etching (formation of vacancy islands), and a surface remaining intact. To assess the impact of chloride and sulfate contaminants, similar experiments were performed in 0.1 M HClO_4_ solutions containing 10 μM H_2_SO_4_ and HCl.

### Oxidation–Reduction Cycles of Au(111) in 0.1 M HClO_4_

**Figure 1:**
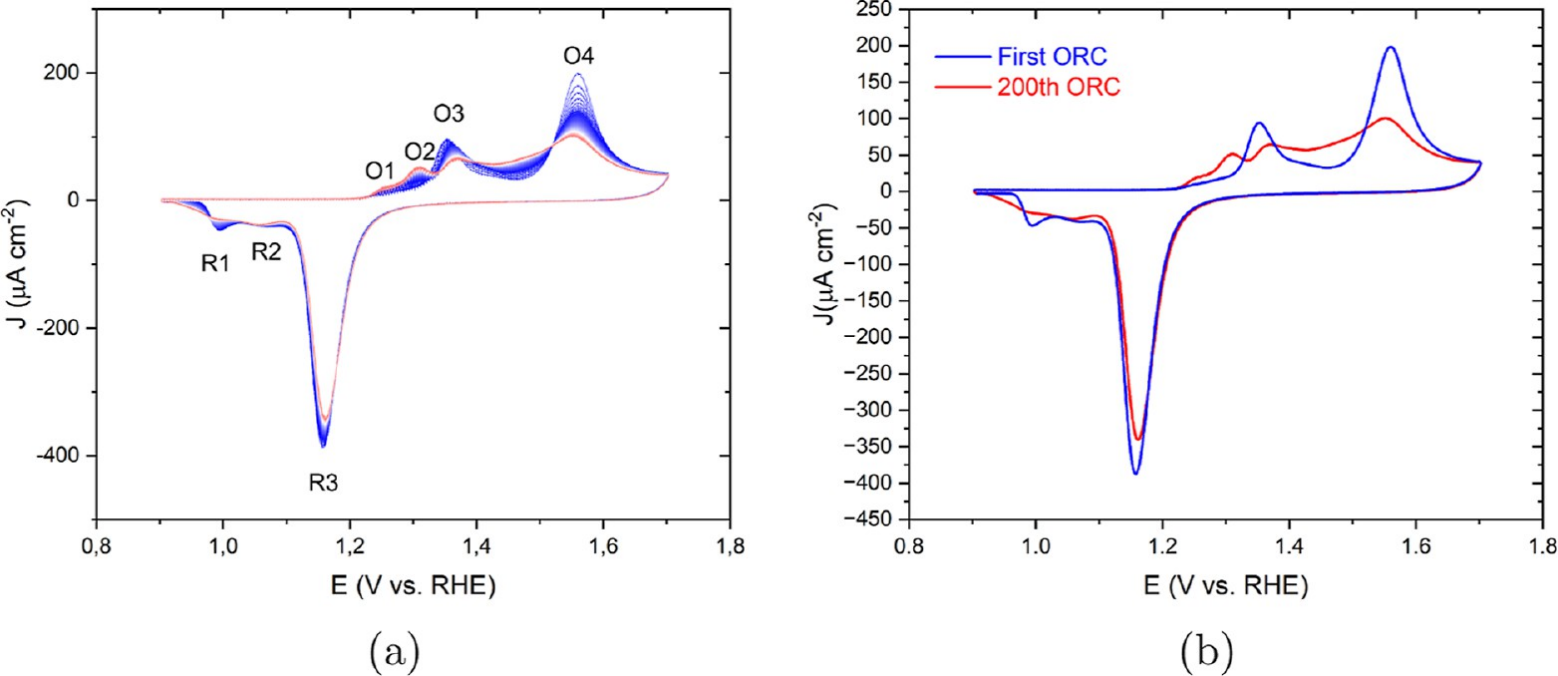
(a) Cyclic voltammograms of the consecutively applied 200 ORCs on Au(111) in 0.1 M HClO_4_ with a scan rate of 50 mV s^–1^ in the potential window of 0.9 to 1.7 V versus RHE from the first (blue) to 200th (red) and (b) only the first (blue) and the 200th (red) ORC.

Figure 1a shows the changes in the cyclic voltammogram of Au(111) as a consequence of the ORCs in a conventional electrochemical cell containing a 0.1 M HClO_4_ solution, scanning from 0.9 to 1.7 V at 50 mV s^–1^. The first cycle can be used as a benchmark for the cleanliness and crystallographic orientation of the single-crystal surfaces.^39,40^ The first CV just after annealing is shown separately and agrees well with the existing literature, together with the final CV after 200 ORCs (Figure 1b). Au(111) shows a double-layer region up to 1.1 V, with negligible or very weak specific adsorption of perchlorate anions.^37^ At higher potentials, the CV features two anodic peaks O3 and O4, respectively, at approximately 1.35 and 1.55 V. Angerstein-Kozlowska et al. have attributed these peaks to the replacement of adsorbed anions with hydroxide (O3) and further gold oxidation accompanied by some remaining anion desorption (O4).^41,42^ Upon cycling, two smaller shoulder-type peaks appear at potentials below the O3 peak called O1 and O2, likely related to OH and O adsorption on low-coordinated sites generated during the oxidation–reduction cycling. Simultaneously, the charge corresponding to the O3 and O4 peaks decreases with an increasing number of cycles, indicating that the (111) terrace is lost during the process. In the negative-going scan, a prominent cathodic peak exists at 1.18 V, labeled as R3, associated with the reduction of the surface oxide.^41,42^ There is a shoulder to R3 at more negative potentials, which has been assigned to the reduction of two sublattices of MOH on an anion-free surface.^41,43^ With cycling, this shoulder develops two separate peaks, R1 and R2. Calculated oxidation and reduction charge densities for the 200 ORCs are listed in Figure S1.

**Figure 2:**
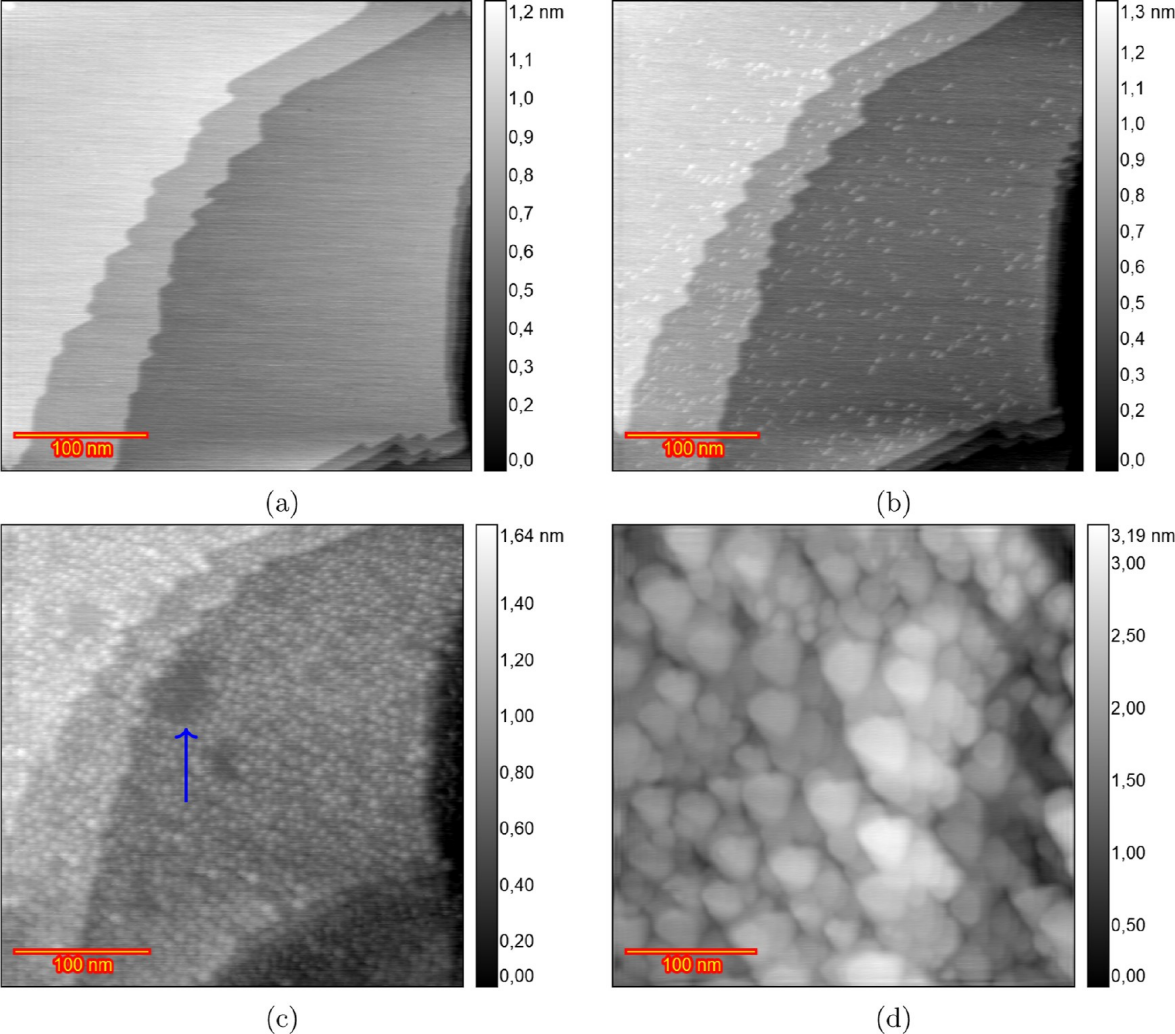
EC-STM image (350 × 350 nm) of Au(111) in 0.1 M HClO_4_. (a) Sample surface at 0.7 V vs RHE just after annealing. (b) Partially lifted reconstruction at 0.9 V. (c) After *n* ORCs from 0.9 to 1.65 V and imaging at 0.9 V *n* = 15 and (d) *n* = 200.

Figure 2 displays EC-STM images of the Au(111) surface in a 0.1 M HClO_4_ solution from the initial reconstructed surface to the roughened surface after 200 ORCs. In the double-layer region at 0.7 V (Figure 2a), the images reveal wide, atomically flat terraces, separated by steps with a monatomic height of 0.23 nm (Figure S2), consistent with the reported 0.236 nm monatomic step height of the Au(111) surface.^32,44^ At 0.9 V (Figure 2b), small monatomic islands begin to form on the terraces due to the lifting of surface reconstruction from  to (1 × 1). Thus, lifting of the reconstruction can take place between 0.7 and 0.9 V. Figure 2c, captured after 15 ORCs from 0.9 to 1.65 V at a scan rate of 50 mV s^–1^, with the potential held at 0.9 V during imaging, shows the Au(111) surface covered with atomic islands on the terraces caused by place exchange during the ORCs. An increase in surface roughness with an increasing number of ORCs was previously observed by Ye et al. using in situ EC-STM (Figure 2 in ref (30)). Although the majority of the surface was roughened, some areas stayed remarkably pristine, as illustrated by the darker areas in Figure 2c pointed out with the blue arrow. Further imaging after applying ORCs for 15, 25, 40, 50, 110, 170, and 200 cycles (25–170 in Figure S3) reveals that the size of the islands increases with the number of cycles, as illustrated in the image Figure 2d obtained after 200 ORCs. As the roughness increases, the darker areas, which initially show no roughening, diminish in size and eventually disappear after approximately 50 cycles. The tendency of forming large islands was consistently observed across three different experiments, as illustrated in Figure S4a after 200 cycles and Figure S4b,c after 70 cycles. There are some inhomogeneities in the size of formed islands after the 200 ORCs, in contrast to the results in sulfuric acid.^38^ This inhomogeneity interferes with the calculation of the height–height correlation function so that no meaningful correlation length and roughness calculation can be obtained.

**Figure 3:**
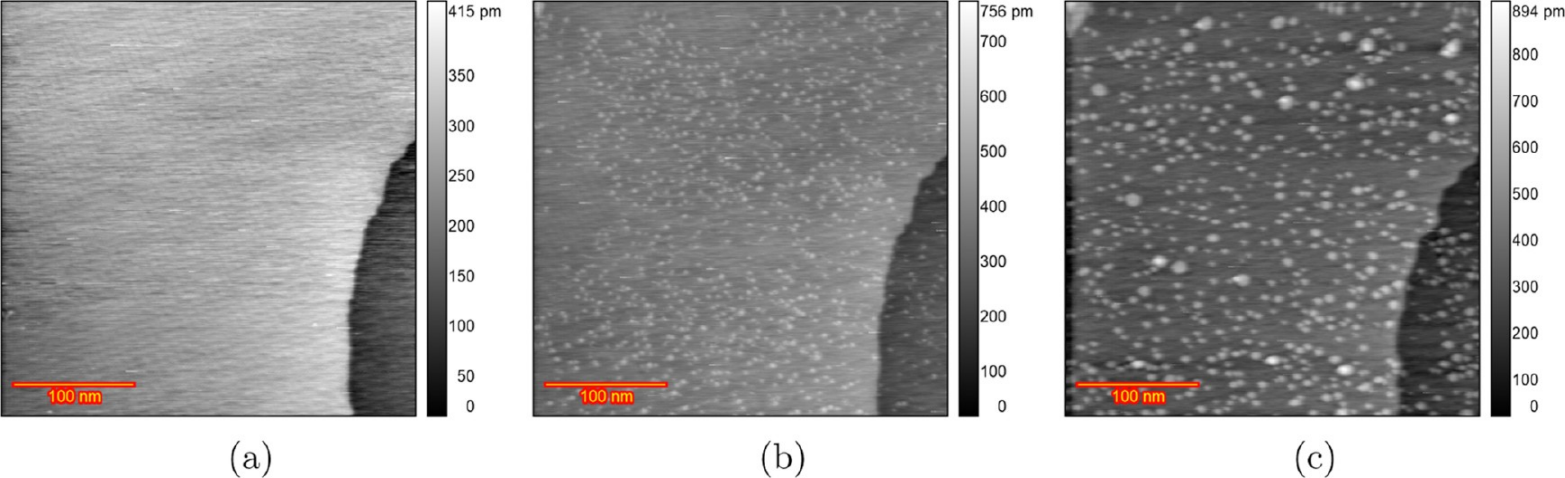
EC-STM image (350 × 350 nm) of Au(111) in 0.1 M HClO_4_. (a) Sample surface at 0.8 V vs RHE just after annealing. (b) Partially lifted reconstruction at 0.9 V. (c) After 13 ORCs from 0.9 to 1.65 V and imaging at 1.1 V.

In multiple experiments, we observed that the surface structure of Au(111) in HClO_4_ solution varied across different experiments, especially after ORCs, as illustrated by the images of an experiment shown in Figure 3. The experiment in that figure was, in principle, the same as the experiment in Figure 2. In the double-layer region at 0.8 V (Figure 3a), the image shows broad, atomically flat terraces with a well-defined herringbone structure and a step line of monatomic step height. At 0.9 V (Figure 3b), small monatomic islands start to form on the terraces due to lifting of the reconstruction. The surface of Au(111) was scanned from 0.9 to 1.65 V at a rate of 50 mV s^–1^, with the potential held at 1.1 V during imaging. Notably, after 1, 5 (Figure S5c,d), and 13 (Figure 3c) cycles, the monatomic islands and step edges remained stable, with the surface largely unchanged except for a few larger islands (∼10 nm in diameter). As a consequence of going through the oxidation–reduction process and place-exchange mechanism, the formation of many adatom and vacancy islands was expected. However, the surface remained unaffected. This behavior was also observed in other experiments (Figure S6). Other experiments were also performed with the remaining potential of 0.9 V prior to this experiment, leading to comparable results, indicating that the remaining (or imaging) potential plays no role in this behavior.

**Figure 4:**
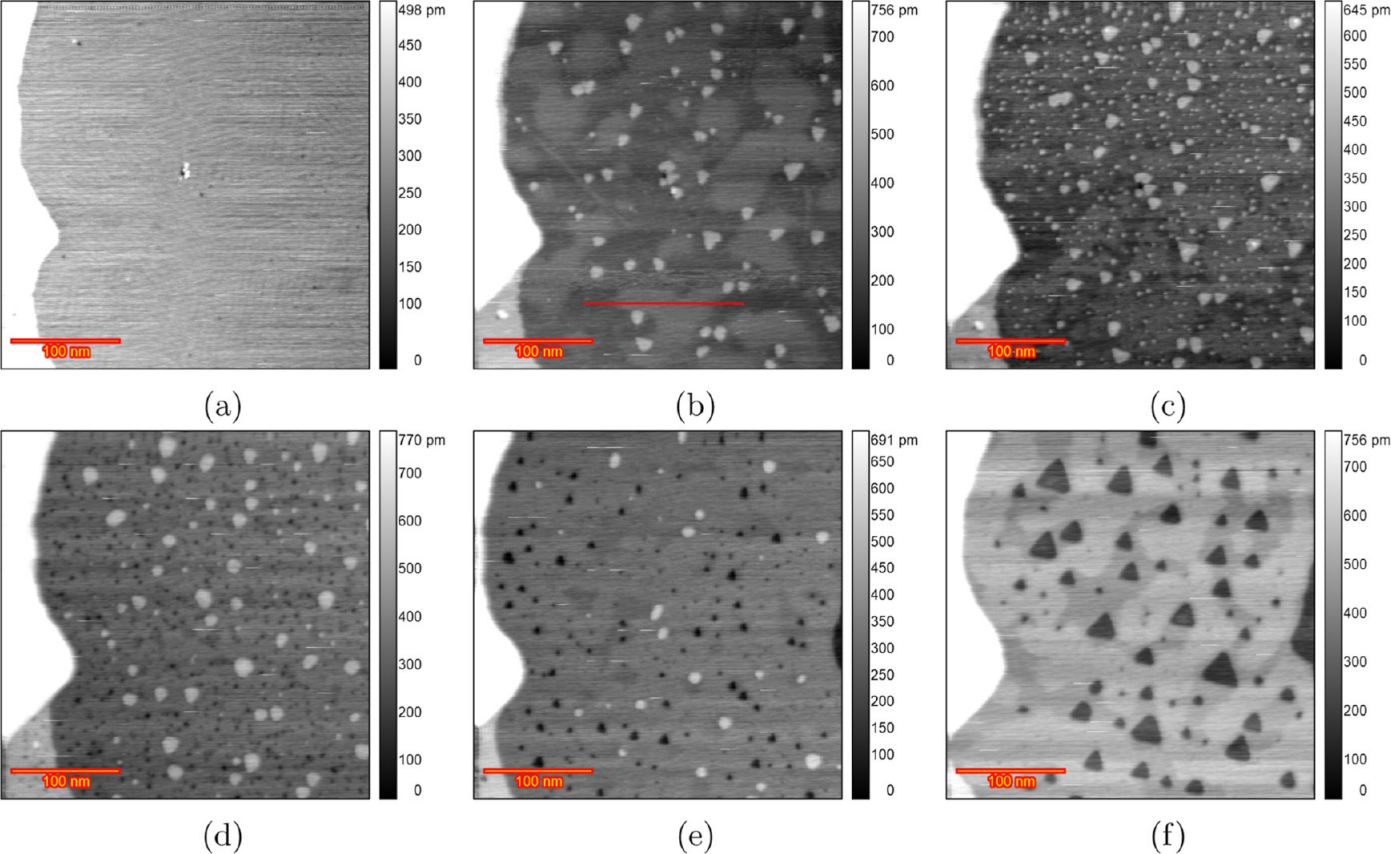
EC-STM image (350 × 350 nm) of Au(111) in 0.1 M HClO_4_. (a) Sample surface at 0.8 V vs RHE just after annealing. (b) Lifted reconstruction at 0.95 V. (c) After *n* ORCs from 0.9 to 1.65 V and imaging at 1.1 V *n* = 1, (d) *n* = 8, (e) *n* = 15, and (f) *n* = 50.

Figure 4 illustrates another distinct behavior of the Au(111) surface structure in a HClO_4_ solution. Figure 4a shows the surface in the double-layer region at 0.8 V with reconstruction. The reconstruction is almost completely lifted at 0.95 V (Figure 4b) and leads to large adatom islands formation. These adatom islands are larger than those observed in the experiments in Figures 2b and 3b. This indicates differences in the early stage of the experiment. After the initial scan from 0.9 to 1.65 V at 50 mV s^–1^, and imaging at 1.1 V, small adatom islands appeared on the terraces (Figure 4c). By the eighth cycle (Figure 4d), small vacancy islands began to grow and the number of the adatom islands reduced. The large adatom islands disappeared by the 15th ORC (Figure 4e), and after 50 cycles, triangular pits formed (Figure 4f). Continued cycling leads to coalescence of the pits and etched terraces sequentially, as shown in Figure S7J,k after 125 and 200 cycles.

**Figure 5:**
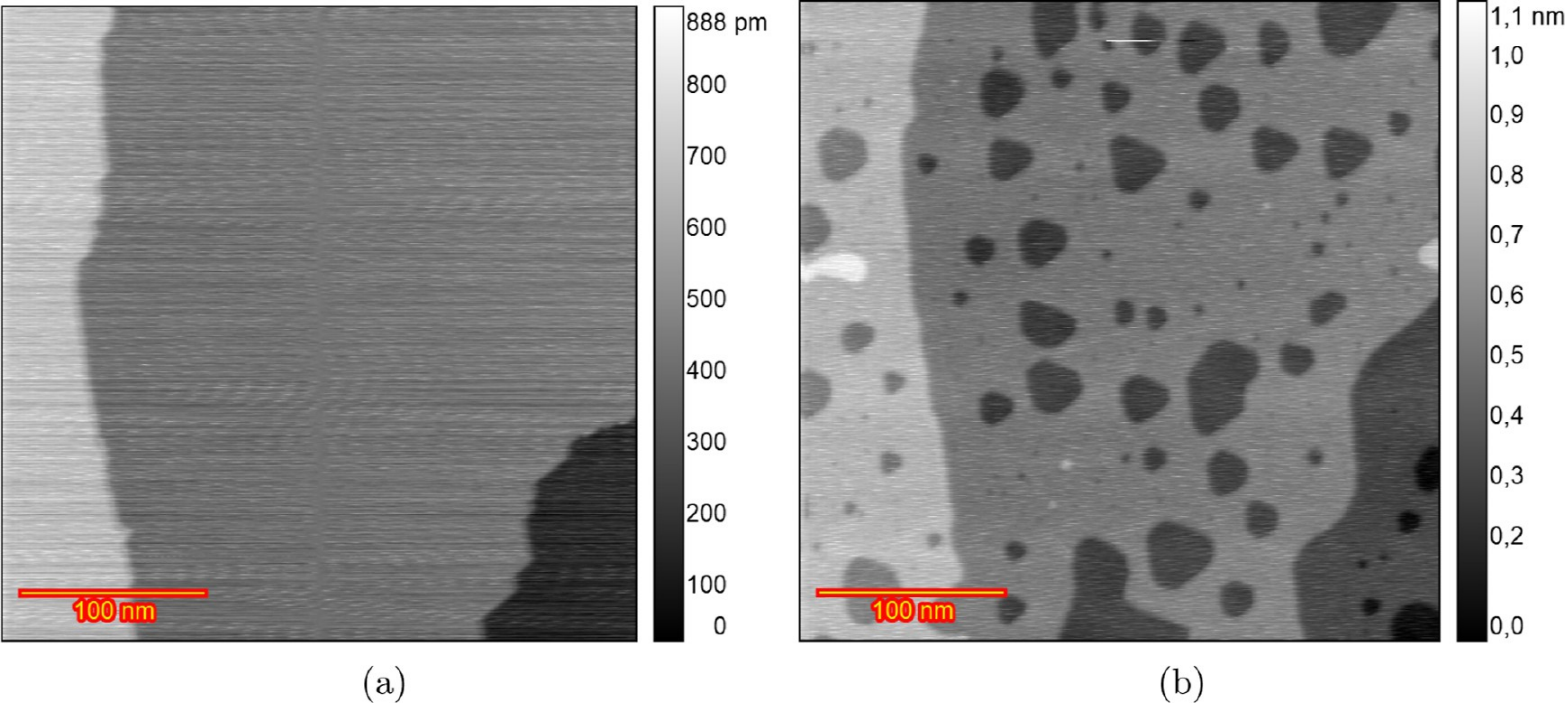
EC-STM image (350 × 350 nm) of Au(111) in 0.1 M HClO_4_ (ROTIPURAN). (a) Sample surface at 0.6 V vs RHE just after annealing. (b) After 100 ORCs from 0.9 to 1.65 V and imaging at 0.9 V.

To check for a possible role of impurities in the electrolytes used in previous experiments and verify the consistency of the results, we utilized a ROTIPURAN Ultra/Supra HClO_4_ electrolyte, renowned for its exceptional purity, to ensure consistent outcomes. Figure 5 presents the results obtained by using this electrolyte. At 0.6 V in the double-layer region (Figure 5a), the surface displayed broad, atomically flat terraces. After the reconstruction was lifted, the terraces were partially covered by islands (Figure S8b). These atomic islands grew in size and number by the second and fifth ORC (Figure S8c,d). Although in the previous experiment, the vacancies began to grow after the eighth ORC, in this case, there is no significant growth of the vacancies after 20 ORCs (Figure S8e). After 30 cycles, vacancies start to grow partially, and the adatom islands disappeared (Figure S8f). Ultimately, as shown in Figure 5b after 100 cycles, the terraces were strongly etched one by one, as observed in Figure 4f. Higher cycle numbers up to 200 ORCs caused more etching, as shown in Figure S8j–l. We suggest two possible explanations for the disappearance of the adislands and the formation of vacancies. The first possibility is that locally adsorbed contaminants, likely chloride, are moved by the scanning tip, altering the surface behavior by modifying the double-layer structure. The second possibility is that initially, the surface was free of impurities, allowing the growth of islands. However, after 20 ORCs, impurities from the solution, again likely chloride, were absorbed onto the gold surface, reducing the built-up roughness and islands and finally etching the surface. It is well-known that perchloric acid may contain anionic impurities.^45^ Therefore, we decided to perform experiments with small amounts of sulfate and chloride deliberately added to the perchloric acid electrolyte. In future research, spectroscopy techniques such as X-ray photoelectron spectroscopy (XPS) could be used to identify specific surface contaminants, although having the necessary spatial resolution will be a challenge. Moreover, higher-resolution scanning tunneling microscopy may lead to unraveling of the double-layer structure in the unchanged areas.

### Role of H_2_SO_4_ Contamination in HClO_4_ Solution on the Au(111) Surface Structure

**Figure 6:**
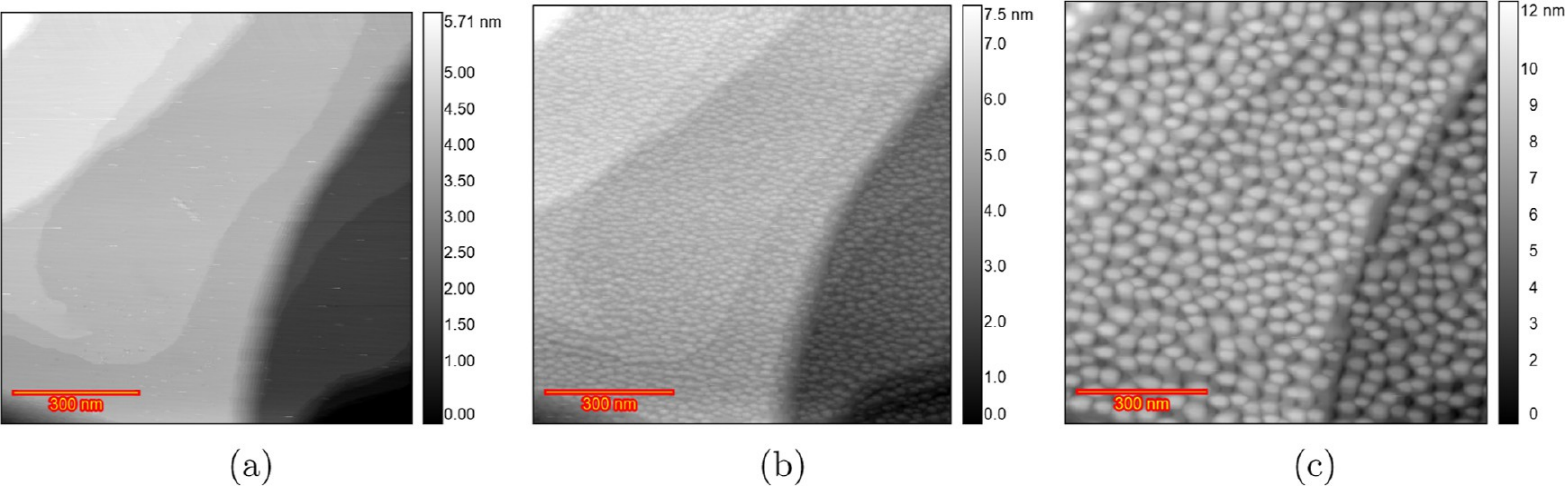
EC-STM image (350 × 350 nm) of Au(111) in 0.1 M HClO_4_ containing 10 μM H_2_SO_4_. (a) Sample surface at 0.0 V vs RHE just after annealing. (b) After *n* = 10 ORCs from 0.8 to 1.65 V and imaging at 0.8 V and (c) *n* = 200.

Since sulfate adsorbs more strongly than perchlorate, we added 10 μM H_2_SO_4_ to 0.1 M HClO_4_ in the EC-STM experiment (Figure 6a) to examine its effect. In the double-layer region (0.0 V), wide atomically flat terraces and approximately straight and curved step lines were visible. Figure 6b displays an image acquired after 10 cycles of potential scanning from 0.8 to 1.65 V at a scan rate of 50 mV s^–1^, with the potential of Au held at 0.8 V during imaging. This observation reveals that the Au(111) surface is completely covered with islands after oxidation and reduction and the step edges remain pristine. Imaging after applying ORCs for 20, 35, 50, 75, 100, 125, 150, 175 (Figure S9), and 200 cycles (Figure 6c) shows an increase in surface roughness. Scanning more than 23 different/random (Figure S10) locations after 200 ORCs confirmed that the surface structure observed in Figure 6c is consistent across the scanned locations. The final texture and level of roughening of the Au electrode in the solution containing 10 μM H_2_SO_4_ (Figure 6c) is similar to that observed in the experiment with pure sulfuric acid (Figures 3 and 5 in ref (38)). A comparison with the pure HClO_4_ solution (Figure 2d) reveals that the size distribution of the islands is more uniform in the experiment with H_2_SO_4_ in solution than that in the pure HClO_4_ experiment. Figure S11 shows the corresponding CVs of the 200 ORCs of the annealed Au(111) in a conventional electrochemical cell containing HClO_4_ solution +10 μM H_2_SO_4_.

**Figure 7:**
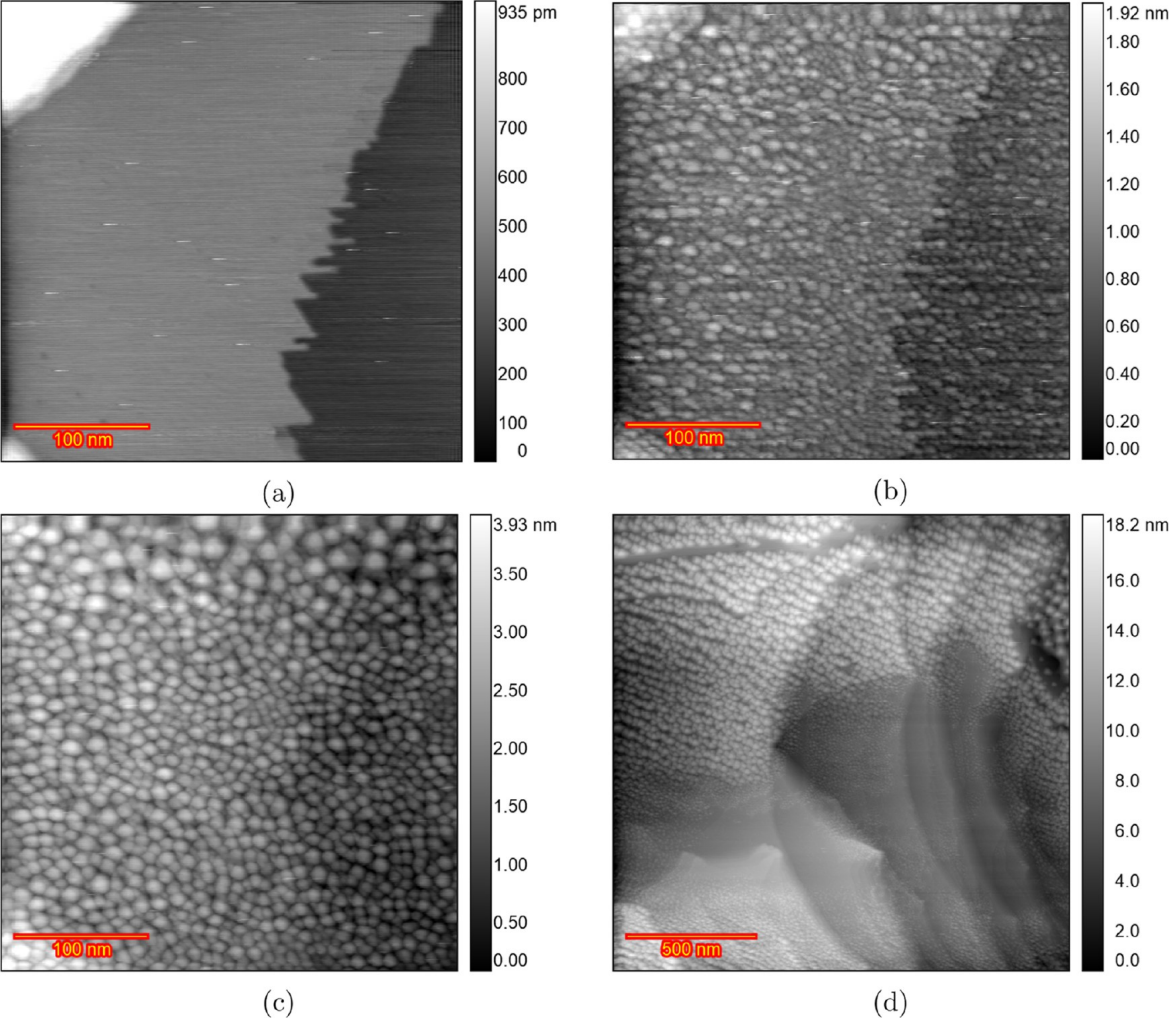
EC-STM image (350 × 350 nm) of Au(111) in 0.1 M HClO_4_ containing 10 μM H_2_SO_4_. (a) Sample surface at 0.0 V vs RHE just after annealing. (b) After *n* = 10 ORCs from 0.8 to 1.65 V and imaging at 0.8 V and (c) *n* = 200 and (d) zoomed out.

In another experiment, under in-principle identical experimental conditions, regions with no roughening were observed. Similar to the experiment described in the previous paragraph, atomically flat terraces were seen at 0.0 V (Figure 7a). After 10 ORCs from 0.8 to 1.65 V at 50 mV s^–1^, with holding the potential at 0.8 V during imaging (Figure 7b), the surface became completely covered with atomic islands. Imaging after 200 ORCs (Figure 7c) showed an increase in the size and height of the islands. However, the top part of the image showed larger islands in comparison to the rest of the image. Surprisingly, zooming out and imaging different locations after 200 ORCs (Figure 7d) revealed areas where no roughening had occurred. Since the step lines are clearly visible at the center of the image, the flat areas cannot be caused by improper surface imaging. This observation indicates that even in the presence of H_2_SO_4_, inhomogeneity in the surface roughening can still be observed after many ORCs. This is likely due to the presence of chloride impurities, which are absorbed more strongly onto the surface than is H_2_SO_4_. As a result, the double layer is not homogeneous across the entire sample surface, leading to variations in surface roughening. This phenomenon was also observed in our previous work,^46^ which examined the effect of small amounts of chloride in H_2_SO_4_. Figure S12 shows the full sequence of experimental images corresponding to Figure 7.

### Role of HCl Contamination in HClO_4_ Solution on the Au(111) Surface Structure

**Figure 8:**
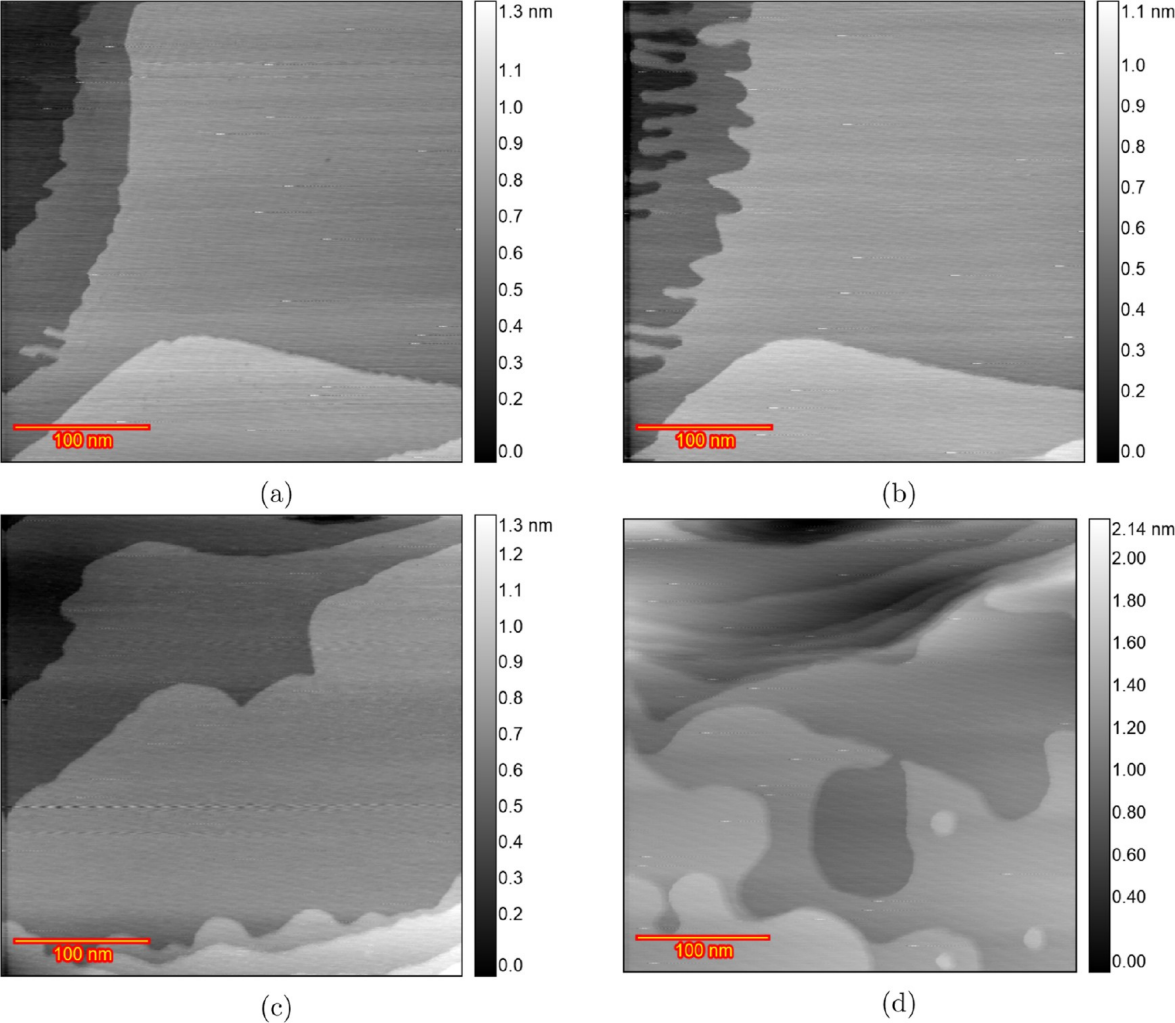
EC-STM image (350 × 350 nm) of Au(111) in 0.1 M HClO_4_ containing 10 μM HCl. (a) Sample surface at 0.0 V vs RHE just after annealing. (b) Lifted reconstruction at 0.9 V. (c) After *n* = 10 ORCs from 0.8 to 1.65 V and imaging at 0.8 V and (d) *n* = 200.

Figure 8 shows EC-STM images of a Au(111) surface in a 0.1 M HClO_4_ solution with 10 μM HCl at various stages during the ORC experiment. At 0.0 V (Figure 8a), wide and atomically flat terraces and monatomic step lines are visible. Figure 8b depicts an image taken at 0.9 V after the lifting of the surface reconstruction. Significant changes in the shape of the step lines were observed compared to the chloride-free solution, although no adatom islands can be seen because of the high mobility of gold atoms in the presence of chloride. Figure 8c presents an image after 10 cycles of potential scanning from 0.8 to 1.65 V at a 50 mV s^–1^ scan rate with the potential held at 0.8 V during imaging. Significant recession of the step lines is observed, with no vacancy islands, and the terraces remain pristine after 10 ORCs. Imaging after 200 ORCs (Figure 8d) shows significant etching and a recession of the step lines. The complete sequence of images for this experiment (Figure 8) is presented in Figure S13. We previously observed that in the presence of chloride in sulfuric acid,^46^ there is significant Au dissolution and the high mobility of gold surface atoms in the chloride-containing electrolyte does not allow the capturing of the vacancies in the STM images. Scanning at various random locations after 200 ORCs confirms that no adatom/vacancy islands can be seen on the scanned areas because of the high mobility of gold atoms in the presence of chloride. This high dissolution rate in chloride-containing electrolytes was repeated in another experiment (Figure S14).

**Figure 9:**
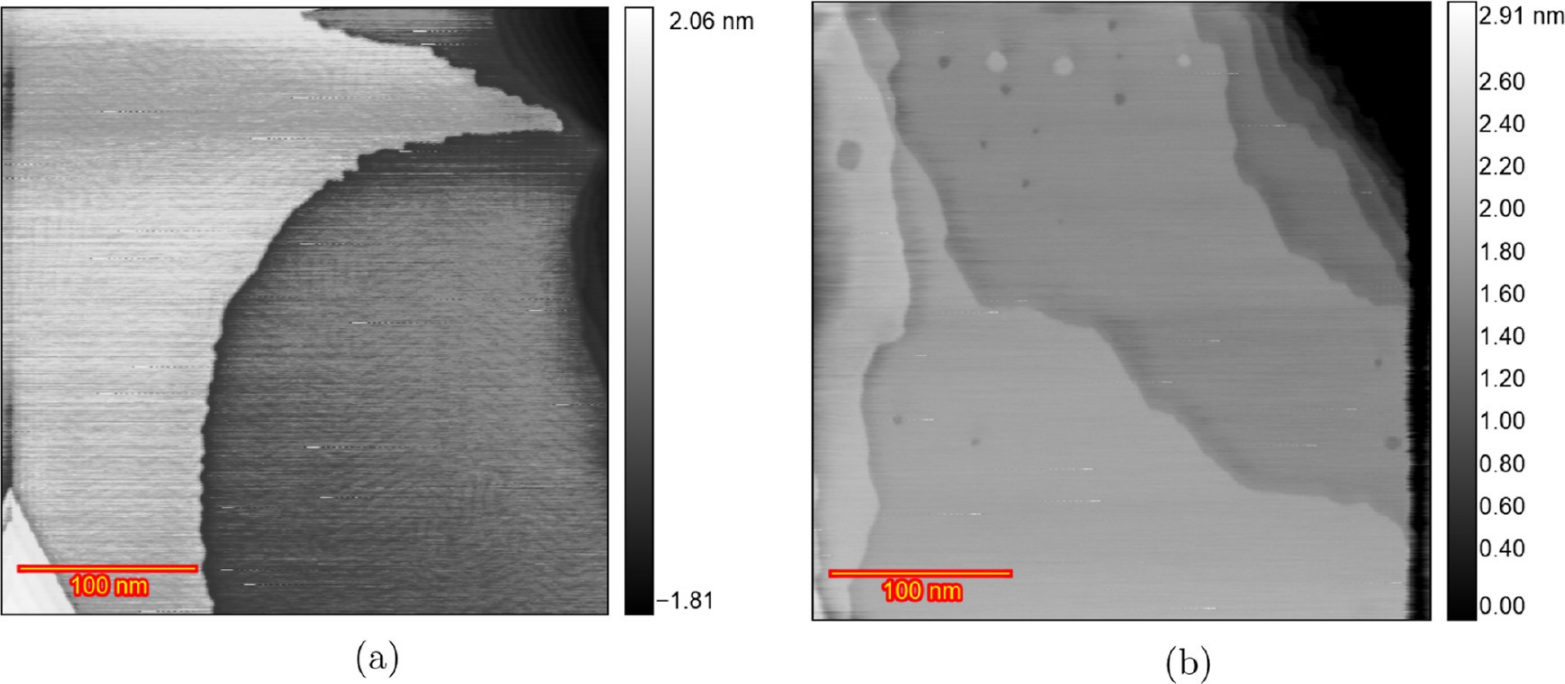
EC-STM image (350 × 350 nm) of Au(111) in 0.1 M HClO_4_ containing 10 μM HCl. (a) Sample surface at 0.0 V vs RHE just after annealing. (b) After *n* = 125 ORCs from 0.8 to 1.65 V and imaging at 0.8 V.

Repetition of the same experiment (as shown in Figure 8) started with a well-defined herringbone reconstruction at 0 V (Figure 9a). Unlike the observations in the experiment described in the previous paragraph, some vacancy islands were captured during sample imaging. For instance, after 125 ORCs (Figure 9b), vacancies are located in the top part of the image. These observations can pinpoint the fact that the extent of chloride adsorption on the sample surface differs even with the additional 10 μM HCl. More images of this experiment are provided in Figure S15.

**Figure 10:**
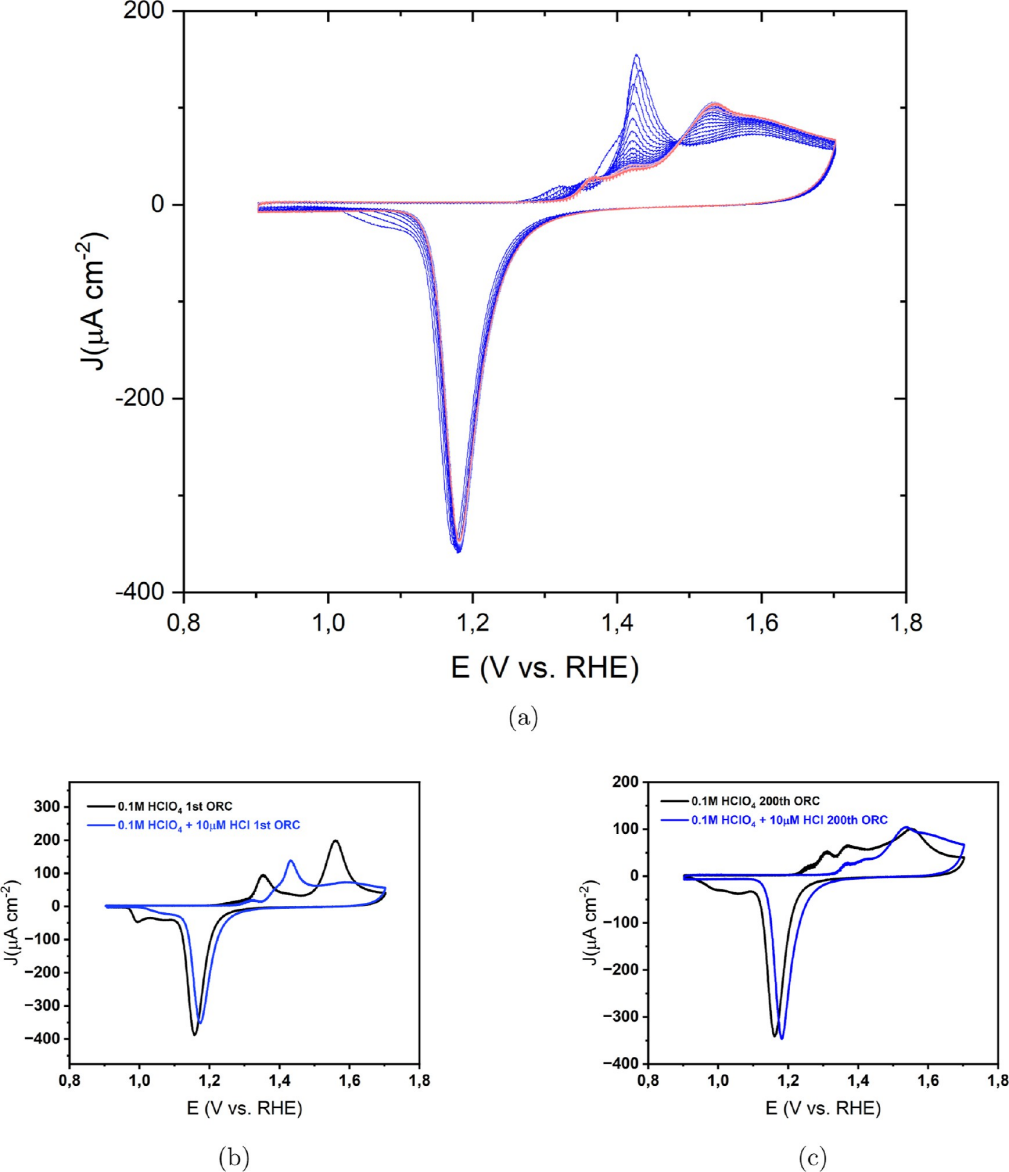
CV of Au(111) in 0.1 M HClO_4_ containing 10 μM HCl in the potential window of 0.9 to 1.7 V versus RHE. (a) All the CVs from the first (red) to 200th (blue). (b) Comparison of the first CV of pure 0.1 M HClO_4_ (black) and the electrolyte containing 10 μM HCl (blue). (c) Comparison of the 200th CV of pure 0.1 M HClO_4_ (black) and the electrolyte containing 10 μM HCl (blue).

Figure 10a shows how the cyclic voltammetric fingerprint of Au(111) changes during cycling from 0.9 to 1.7 V at 50 mV s^–1^. In the initial cycle of the chloride-containing solution (blue curves in Figure 10a), the peaks of OH adsorption and gold oxide formation merge into a single peak around 1.42 V (blue curve in Figure 10b). A previous EQCM study^23^ attributed this anodic peak to the three-electron oxidative dissolution of gold Au + 4Cl^–^ → AuCl_4_^–^ + 3e^–^. Furthermore, as reported in the literature,^31,32^ the reduction peak of the oxide layer shifts to a slightly more positive potential. In subsequent oxidation–reduction cycles, the charge of the peak due to the formation of AuCl_4_^–^ decreases and completely disappears after approximately 15 cycles (Figure 10a), likely due to the decreasing presence of chloride on the surface promoting AuCl_4_^–^ formation. Moreover, after 200 cycles, the peak for gold oxide formation (O4) broadens, as shown in Figure 10c. Calculated oxidation–reduction charge densities for these CVs are listed in Figure S16.

### General Discussion

The EC-STM studies described above demonstrate three primary surface responses of the Au(111) electrode in 0.1 M HClO_4_ solution during ORCs: roughening (formation of adatom and vacancy islands), etching (formation of pits), and surface stability (i.e., the surface remaining largely intact). Our findings demonstrate that the variation in these behaviors must be attributed to trace impurities, most likely chloride.

#### Roughening by Island Formation

The formation of adatom islands and vacancy islands is indicative of surface roughening, which can be observed in both HClO_4_ and H_2_SO_4_ solutions by applying ORCs. This structure is attributed to the place-exchange process that occurs during ORCs, which causes surface atoms to be displaced and rearranged, leading to an increase in surface roughness.^47^ The addition of sulfate impurities to the HClO_4_ solution, being more adsorptive than perchlorate, tends to occupy sites on the surface, as evidenced by changes in the cyclic voltammetry peaks in Figure S11. This apparently leads to a more homogeneous surface roughening, as is evident in Figure 6 where the presence of 10 μM H_2_SO_4_ results in more uniform island sizes and a consistent increase in the surface roughness.

#### Etching and Pit Formation

Contrasting with the roughening behavior, etching is characterized by the formation of pits on the Au(111) surface. The pits begin to form and coalesce into larger ones, as shown in Figures 4 and 5. It is known that 10 μM HCl in 0.1 M sulfuric acid can cause a slow (2 atomic layers over 200 ORCs) gold dissolution, and increasing the chloride concentration leads to increase in dissolution rate and gold atom surface mobility.^46^ Chloride ions are known to strongly adsorb on Au(111), forming a chloride adlayer that can cause gold dissolution through the formation of gold chloride complexes (e.g., AuClO_4_^–^).^48^ This trace level of chloride impurities in perchloric acid appears to lead to strong differences in local chloride absorption and causes gold roughening, the extent of which can vary widely over the surface.

#### Intact Surface

In some cases, the Au(111) surface remains relatively stable with minimal roughening or etching observed. A similar behavior as depicted in Figure 3 was observed in our previous study, in which a very low chloride concentration (1 μM HCl) in 0.1 M sulfuric acid could apparently form an adlayer which blocks surface oxidation–reduction reactions in that area, and consequently, no change in surface structure or surface roughening was observed even after 200 ORCs.^46^ Thus, the very low concentration of chloride can be related to the intact surface over many ORCs. Moreover, the areas that stayed unchanged over ORCs in 0.1 M HClO_4_ are easier to find compared to the experiment with the additional 10 μM sulfuric acid. This indicates that there is a competition between the anions to cover the surface, and this may reduce the intact areas in the presence of sulfate.

**Figure 11:**
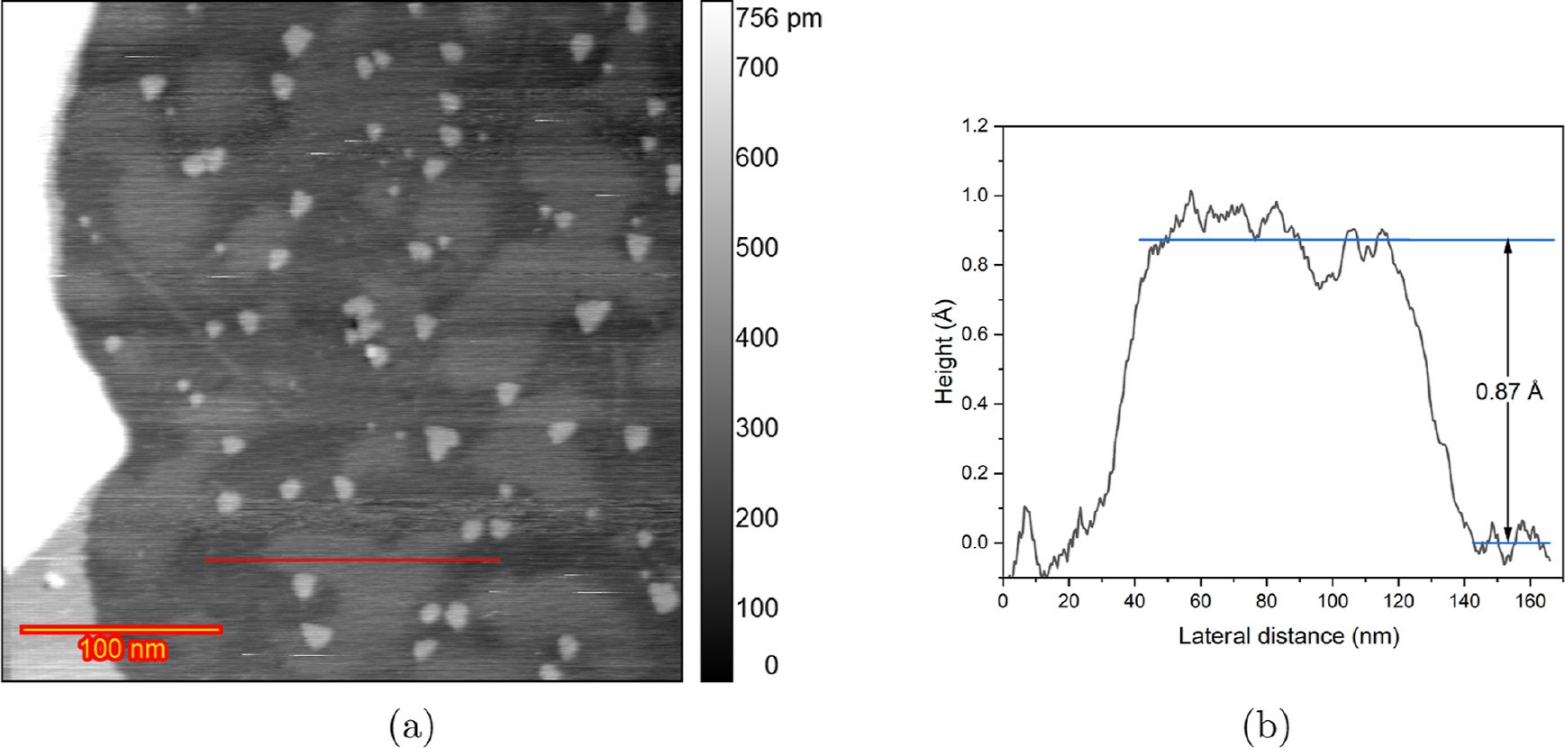
(a) The STM image showed in Figure 4b with the red line indicating the location of the extracted height profile and (b) the corresponding height profile showing the height difference of the higher and lower area on the same terrace.

The observed height changes on the terraces in Figure 4b,f can be explained by the localized adsorption of impurities (most likely chloride) on the surface. At sufficiently high potentials (around 0.9 V vs RHE in this case), anion adsorption occurs, leading to the lifting of the reconstruction. This adsorption can modify the tunneling medium, which in turn can affect the work function. Such changes can alter the magnitude of the tunneling current. Since all images are captured in constant current mode, the feedback system compensates for this change by adjusting the tip height. Consequently, variations in the work function may appear as depressions or protrusions on the surface. Similarly, shadow-like regions on the terraces, as seen in Figure 4b,f, were noted to have heights lower than an atomic step (0.87 Å according to the height profile plot in Figure 11) with indistinct boundaries. Thus, these height variations do not represent the actual topographical features of the sample.

Similar behavior was observed for 1 μM HCl in 0.1 M sulfuric acid.^46^ The impact of the change in the adlayer on the topographical image of Cu(111) in 0.1 M NaOH was observed as a reduced height on the terrace as well (approximately 0.05 nm).^48^ This observation provides some more evidence that the double-layer composition at high enough potentials can be/become inhomogeneous. The observations in Figure 5 suggest that the absorbed layer is not static and can change during the experiment, likely due to interactions with the scanning tip or the diffusion of contaminants in the electrolyte solution. Comparison of Figures 8 and 9b would show some variations in chloride concentration since previous in situ STM studies of Au(111) have shown that the presence of Cl^–^ ions significantly enhances surface diffusion and accelerates annealing on Au(111).^28,31,32^ A similar inhomogeneity in the double-layer composition was also observed in 0.1 M sulfuric acid plus 1 μM HCl in our previous study.^46^

## Conclusions

This study underscores the significant impact of trace impurities on the electrochemical behavior and surface morphology of Au(111) in HClO_4_ solutions, highlighting their role in dictating anisotropic surface evolution. The adsorption of impurities such as chloride alters the local electrochemical environment, influencing the surface oxidation and reduction reactions. These findings emphasize the complexity of the electrochemical double-layer structure and the need to consider impurity effects in both fundamental and applied research studies involving gold electrodes. Understanding these mechanisms is crucial for addressing degradation in noble metals like gold, particularly in environments where maintaining ultrapure conditions is challenging. Further research is essential to elucidate the mechanistic pathways of surface changes and develop strategies for mitigating impurity-induced degradation, thereby improving the performance and longevity of gold-based electrochemical systems.

## Supporting Information Available

Calculated oxidation and reduction charge densities, EC-STM images, CVs, and full-sequence EC-STM images (PDF)
