# Supplementary material for: Anisotropic Roughening of a Au(111) Single-Crystal Electrode Surface in HClO4 Solution during Oxidation–Reduction Cycles
Source: J Phys Chem C Nanomater Interfaces. 2025 May 5;129(19):8915–26. doi: 10.1021/acs.jpcc.5c01177 (PMC12086851; doi:10.1021/acs.jpcc.5c01177)
Supplement: Supplementary file 1 — jp5c01177_si_001.pdf [file jp5c01177_si_001.pdf]

Supporting Information to: Anisotropic Roughening of Au(111) Single-Crystal Electrode Surface in $HClO_4$ Solution during Oxidation-Reduction Cycles

Saeid Behjati,† Mojtaba Hajilo,‡ Maximilian Albers,† and Marc T.M. Koper*,†

†*Leiden Institute of Chemistry, Leiden University, PO Box 9502, 2300 RA Leiden, the Netherlands*

‡*Chemistry Department, Sharif University of Technology, P.O. Box 11155-3615, Tehran 19166, Iran*

E-mail: m.koper@lic.leidenuniv.nl

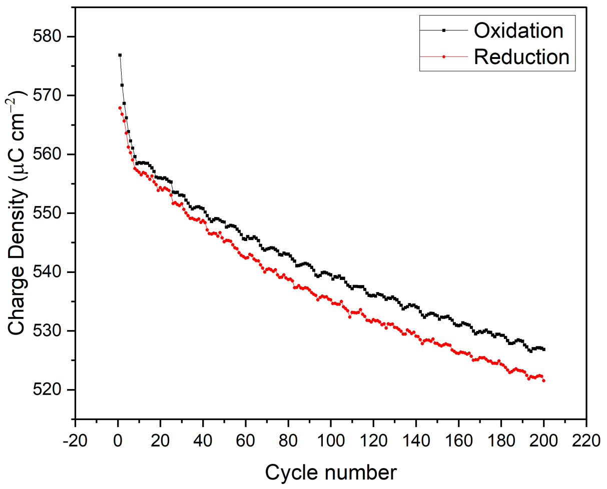

(a)

Figure S 1: Calculated oxidation and reduction charge densities for Au(111) in 0.1 M $HClO_4$ for 200 ORCs.

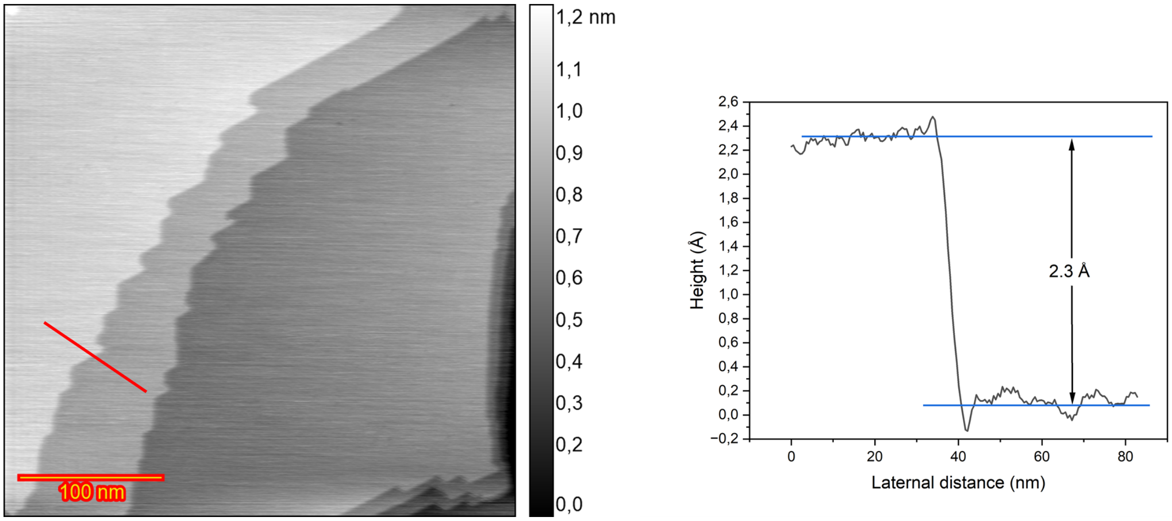

(a)

Figure S 2: EC-STM image (350×350 nm) of Au(111) in 0.1 M $HClO_4$. a) Sample surface at 0.7 V vs RHE just after annealing b) the corresponding height profile of the indicated red line in (a) that shows the step height for Au(111)

In the experiment shown in Figure S3e-g, there is some evidence of a tip-shape effect. Thus, this effect should be considered in the discussions and conclusions for the mentioned images. However, it does not influence the final broader conclusions.

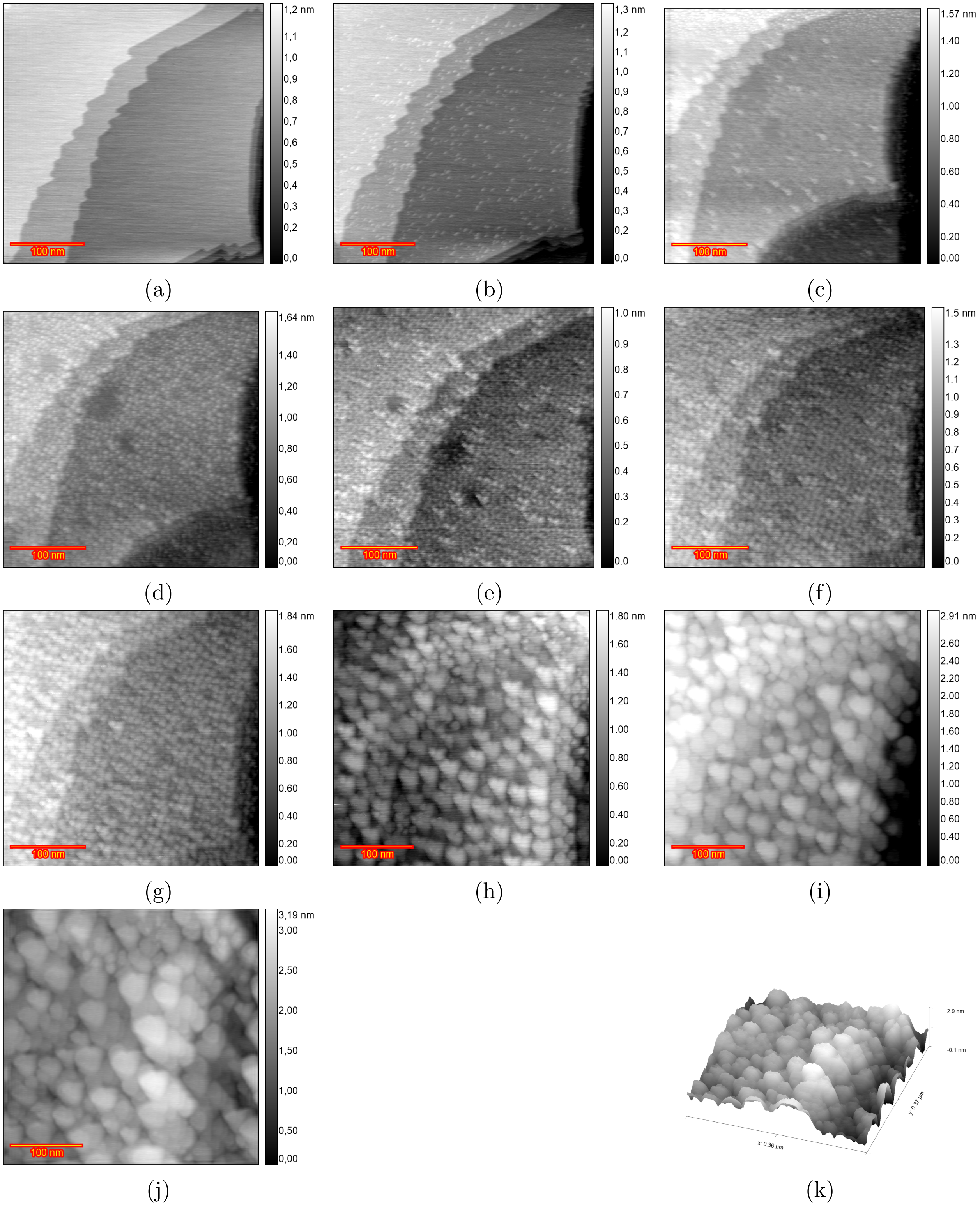

Figure S 3: EC-STM image (350×350 nm) of Au(111) in 0.1 M $HClO_4$. a) Sample surface at 0.7 V vs RHE just after annealing. b) partially lifted reconstruction at 0.9 V. c) after n ORCs from 0.9 to 1.65 V and imaging at 0.9 V n=5 d) n=15 e) n=25 f) n=40 g) n=50 h) n=110 i) n=170 j) n=200 k) 3D image after 200 ORCs.

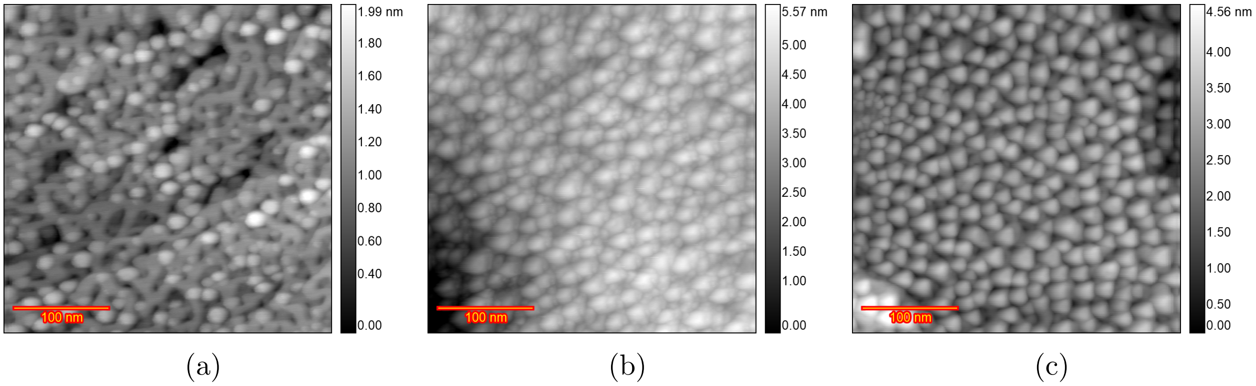

Figure S 4: EC-STM image (350×350 nm) of Au(111) in 0.1 M $HClO_4$ for three different experiment. a) after 200 b,c) after 70 oxidation-reduction cycle.

In the experiment shown in Figure S5, increasing the upper potential limit to 1.8 V after the 13th ORC was tested and led to the formation of some new islands in the bottom right of the image in Figures S5g and S5h. This observation needs more investigation since this indicates the possibility of an unexpected inhibition of the surface oxidation reaction in these areas.

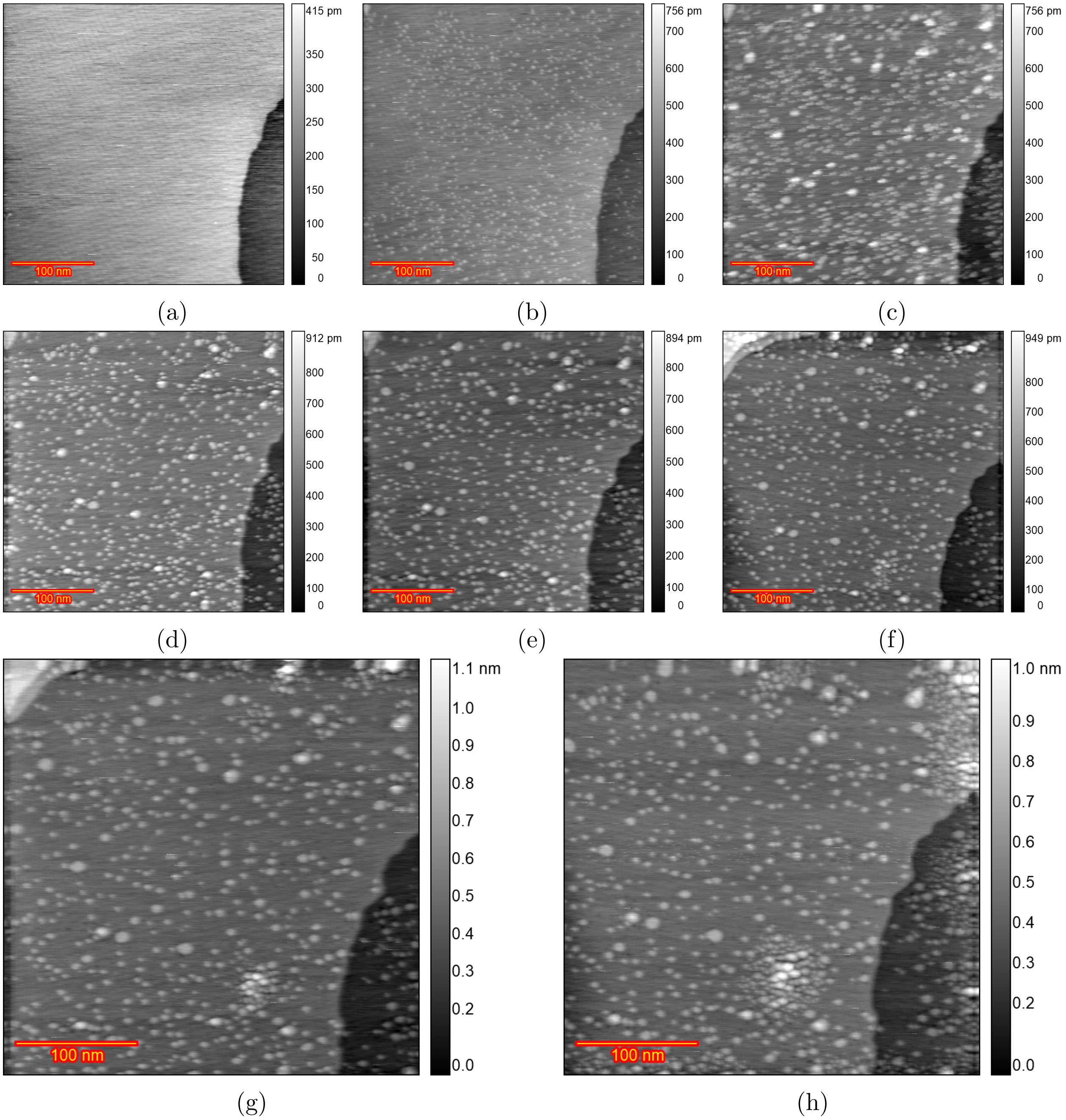

Figure S 5: EC-STM image (350×350 nm) of Au(111) in 0.1 M $HClO_4$. a) Sample surface just after annealing. b) lifted reconstruction at 0.9 V. c) after n ORCs from 0.9 to 1.65 V and imaging at 0.9 V n=1 d) n=5 e) n=13 f) n=15 g) n=20 h) n=30.

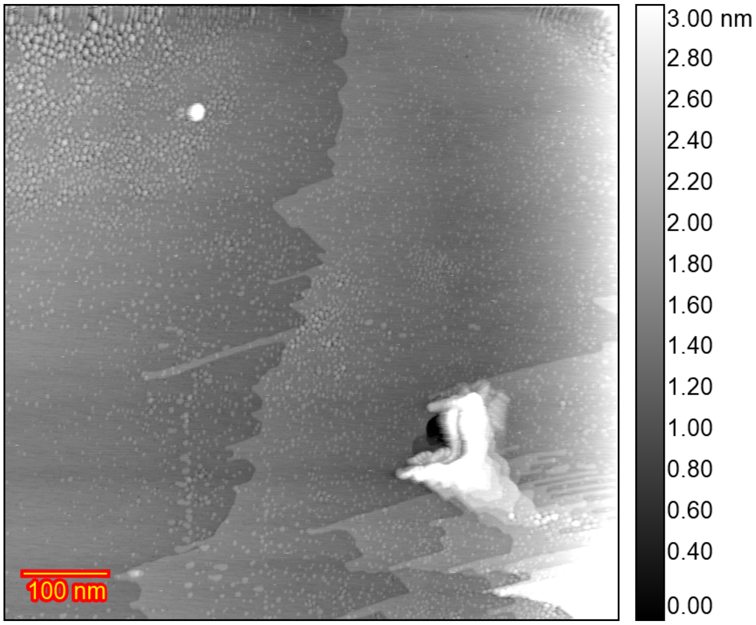

(a)

Figure S 6: EC-STM image (700×700 nm) of Au(111) in 0.1 M $HClO_4$. after 40 cycles. the surface remained largely unchanged except for a few areas.

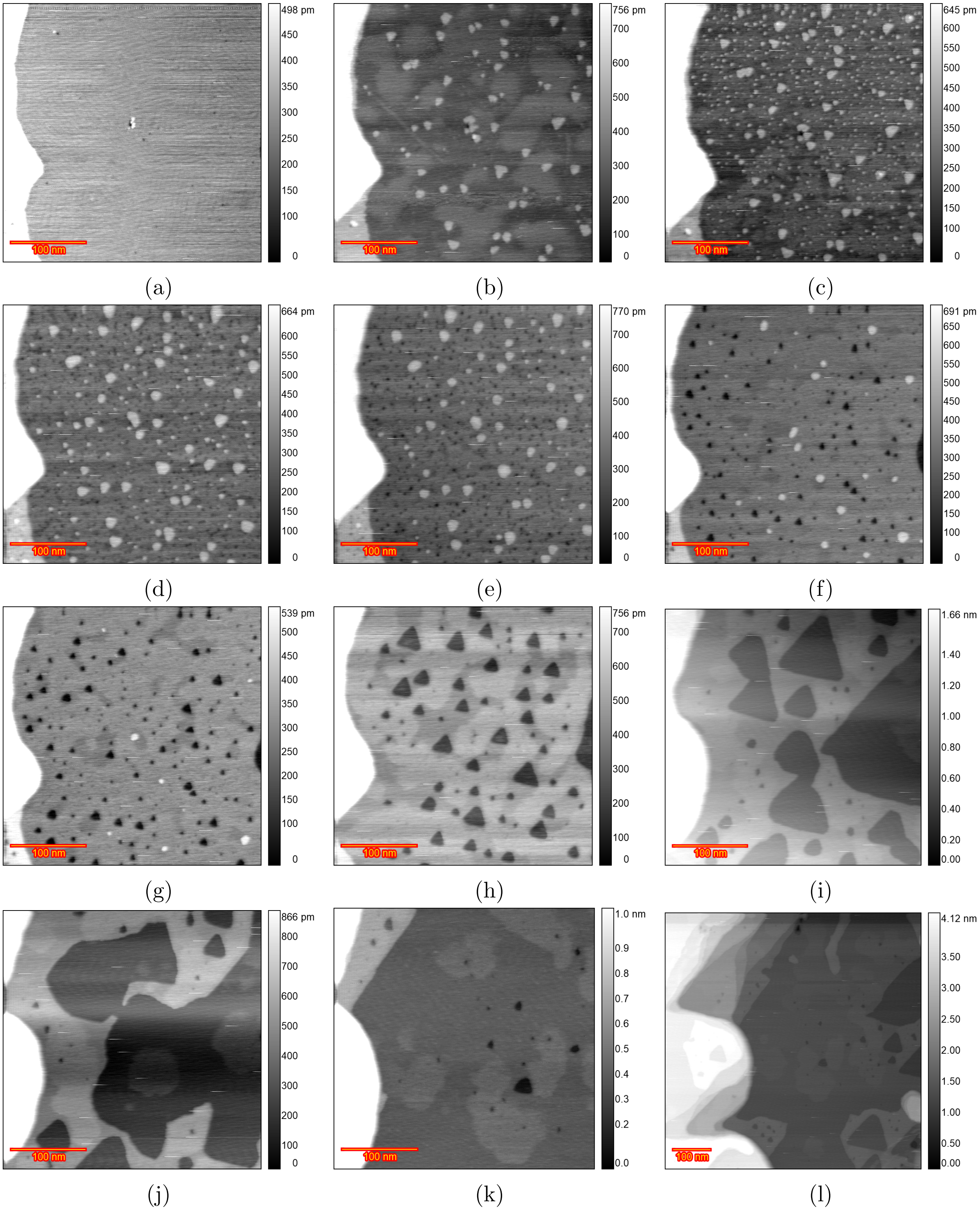

Figure S 7: EC-STM image of Au(111) in 0.1 M $HClO_4$. a) Sample surface just after annealing. b) lifted reconstruction at 0.95 V. c) after n ORCs from 0.9 to 1.65 V, n=1,d) n=5, e) n=8, f) n=15, g) n=20, h) n=50, i) n=125, j) n=150, k) n=200, l) zoomed out after 200 ORCs.

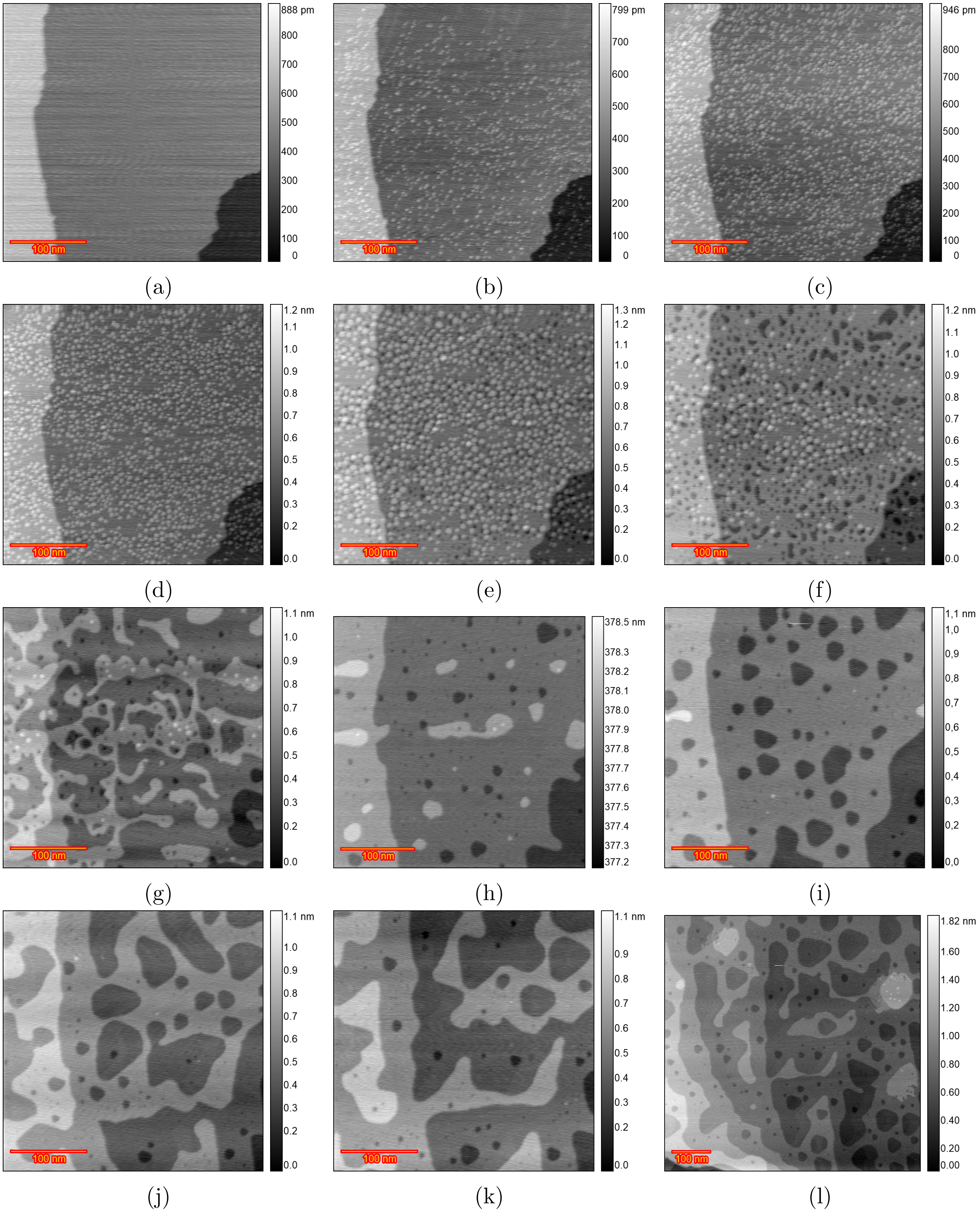

Figure S 8: EC-STM image of Au(111) in 0.1 M $HClO_4$ (ROTIPURAN), a) Sample surface just after annealing, b) lifted reconstruction at 0.9 V, c) after n ORCs from 0.9 to 1.65 V, n=2, d) n=5, e) n=20, f) n=30, g) n=50, h) n=70, i) n=100, j) n=150, k) n=175, l) n=200 zoomed out.

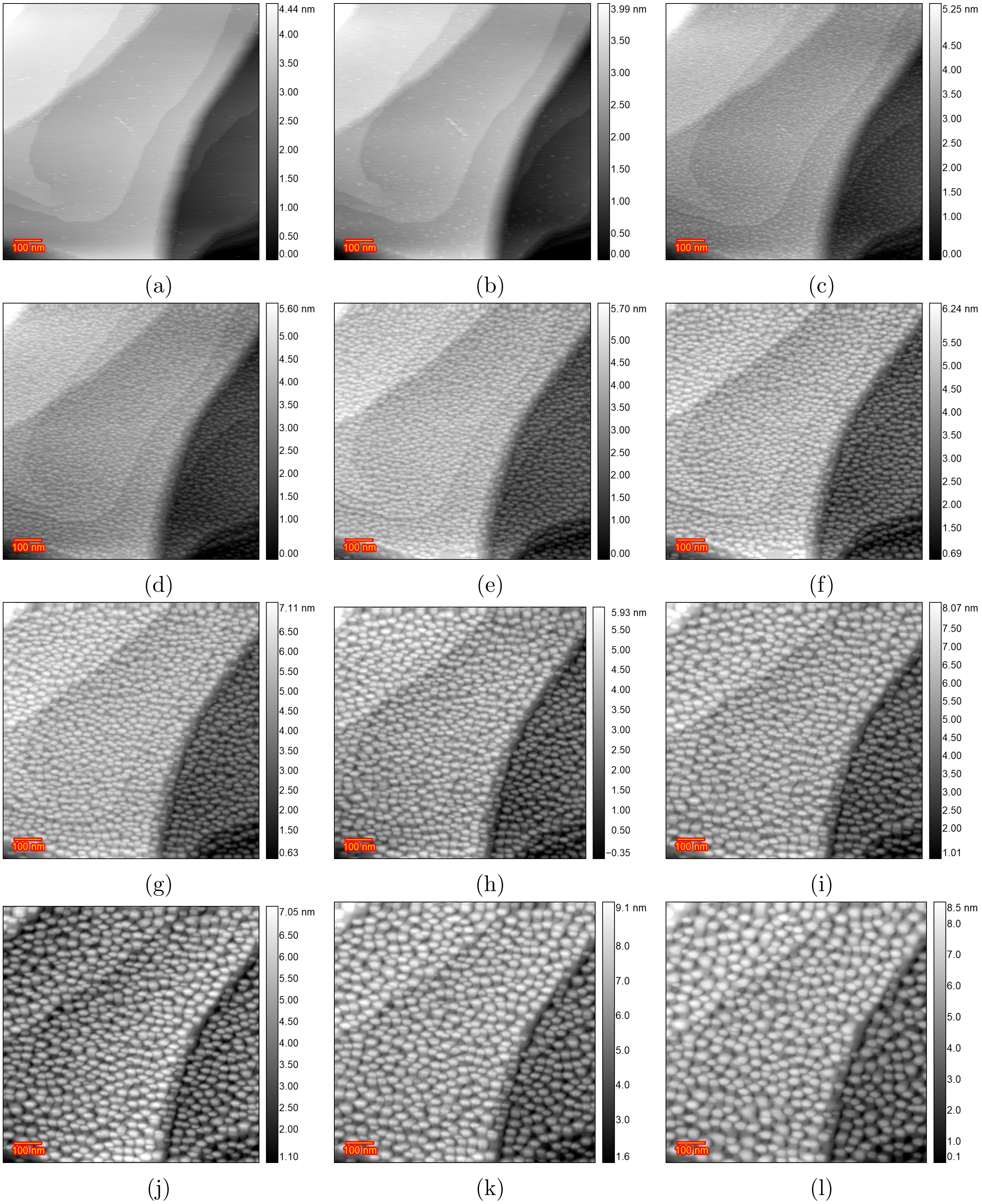

Figure S 9: EC-STM image of Au(111) in 0.1 M $HClO_4$ and 10 µM H_2SO_4, a) Sample surface just after annealing, b) lifted reconstruction at 0.9 V, c) after n ORCs from 0.8 to 1.65 V, n=10, d) n=20, e) n=35, f) n=50, g) n=75, h) n=100, i) n=125, j) n=150, k) n=175, l) n=200.

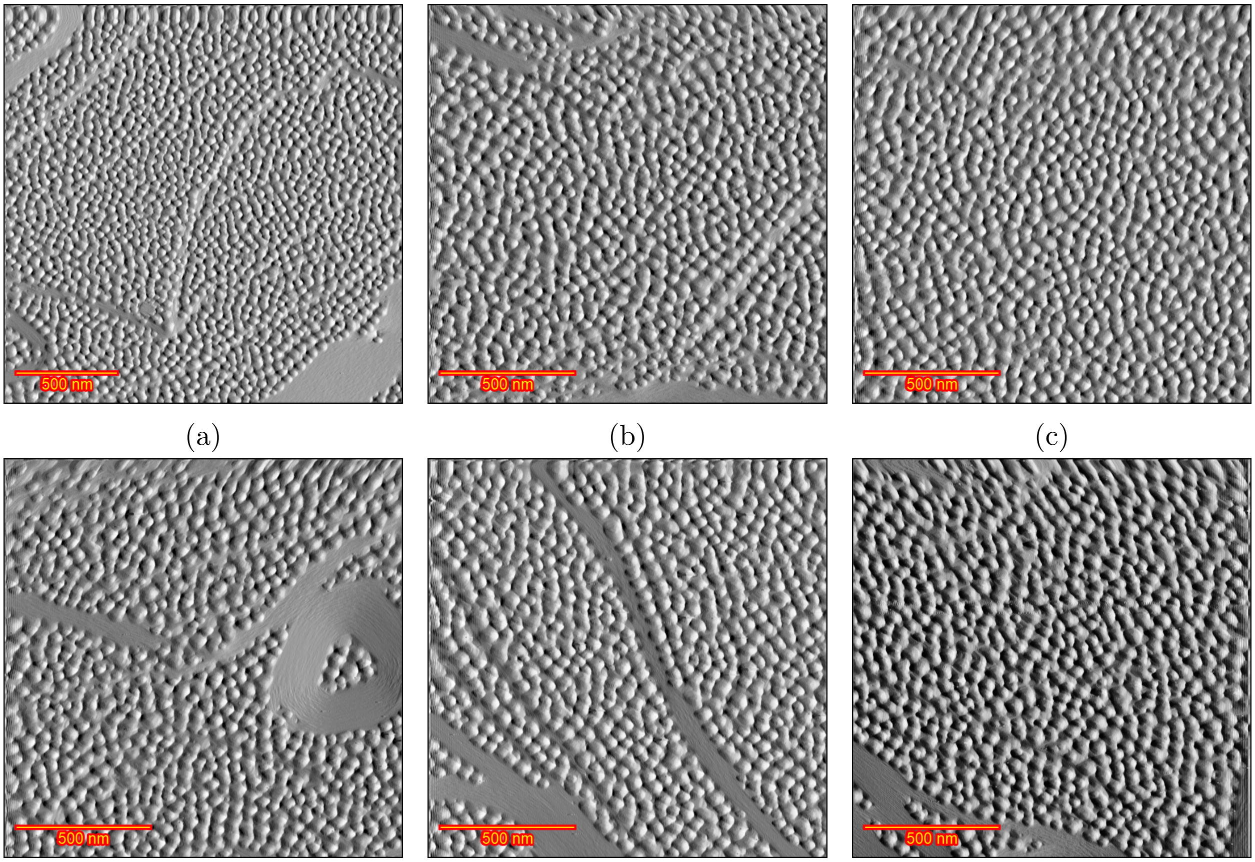

(a) (b) (c)

(d) (e) (f)

Figure S 10: Sequence of EC-STM images of Au (111) at different/random locations after 200 ORCs in 0.1 M $HClO_4$ and 10 µM H_2SO_4 in differential mode.

Figure S11a shows the corresponding CVs of the 200 ORCs of the annealed Au (111) in a conventional electrochemical cell containing $HClO_4$ solution + 10 µM H_2SO_4, scanning from 0.9 to 1.7 V at 50 mVs^{-1} from the first cycle (in blue) to the 200th cycle (in red). Comparing the CVs after the first oxidation-reduction cycle in the H_2SO_4-containing electrolyte and pure $HClO_4$ (Figure S11b), shows an increase in oxide formation (O4) and decrease in OH adsorption (O3) peak in the sulfate-containing solution. This can be explained by the blocking effect of absorbed sulfate on the surface at positive potentials, which blocks the chemisorption of OH^-.[1,2] Further oxidation-reduction cycles lead to a decrease in both O4 and O3 peaks, while the O3 peak completely disappears after 200 ORCs for the sulfate containing electrolyte (Figure S11c). The O2 and O1 peaks become more visible with the

roughness increase (Figure S11a). Figure S11d shows oxidation reduction charge density for the 200 ORCs.

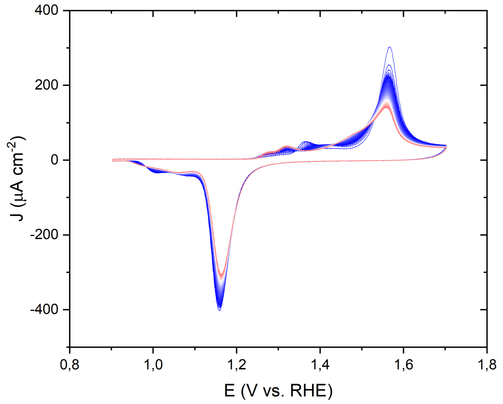

(a)

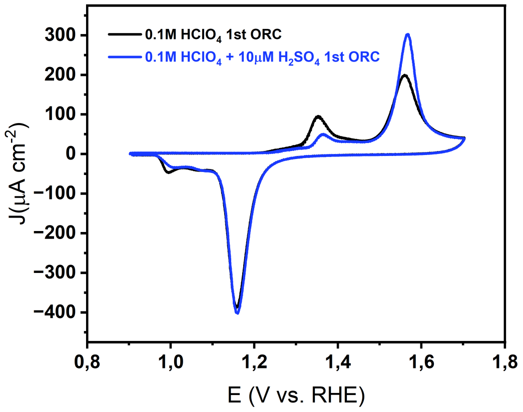

(b)

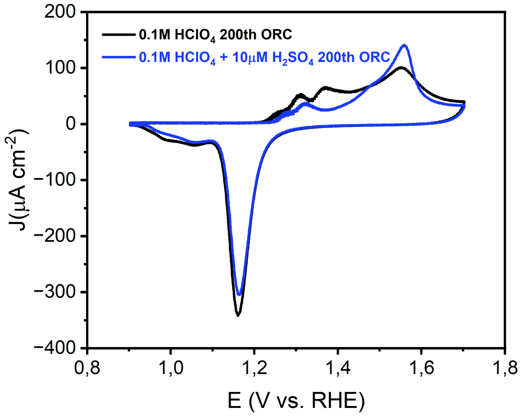

(c)

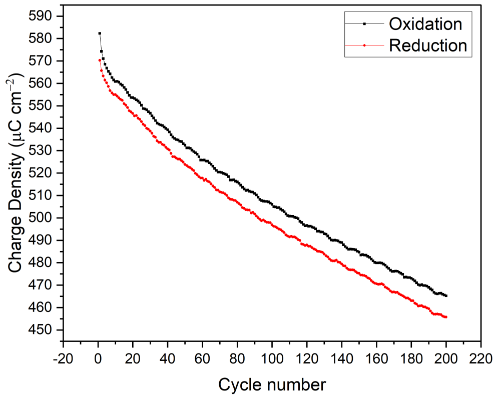

(d)

Figure S 11: CV of Au(111) in 0.1 M $HClO_4$ containing 10 µM H_2SO_4 in the potential window of 0.9 to 1.7 V versus RHE. a) All the CVs from the first (Blue) to 200th (red). b) comparison of the first CV of pure 0.1 M $HClO_4$ (black) and the electrolyte containing 10 M H_2SO_4 (blue). c) comparison of the 200th CV of pure 0.1 M $HClO_4$ (black) and the electrolyte containing 10 M H_2SO_4 (blue). d) Calculated oxidation and reduction charge densities for 200 ORCs.

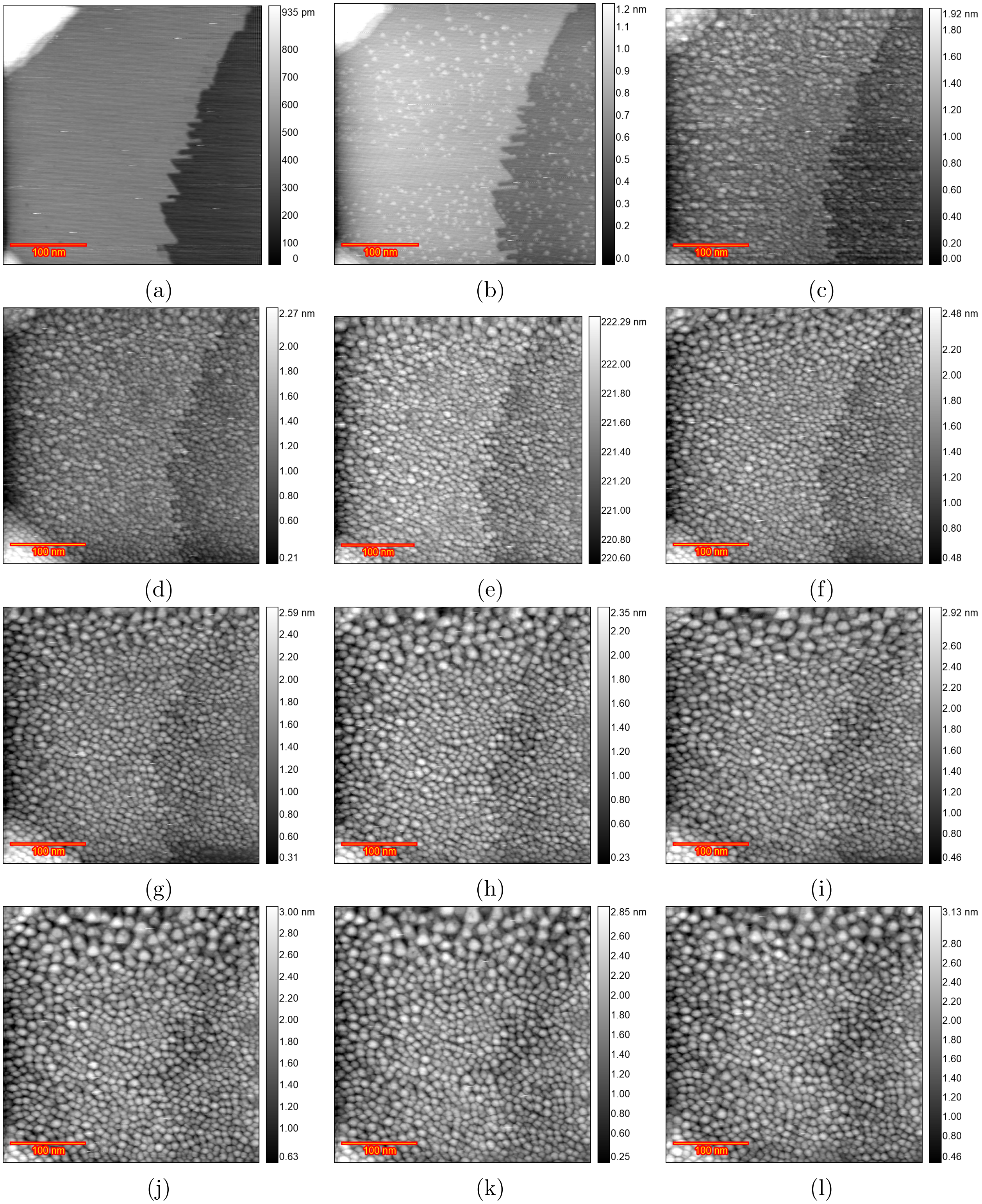

Figure S 12: Full sequence EC-STM images (350×350 nm) of experiment in 0.1 M $HClO_4$ containing 10 µM H_2SO_4. a) Sample surface just after annealing, b) lifted reconstruction at 0.9 V, c) after n ORCs from 0.8 to 1.65 V, n=10, d) n=20, e) n=35, f) n=50, g) n=75, h) n=100, i) n=125, j) n=150, k) n=175, l) n=200.

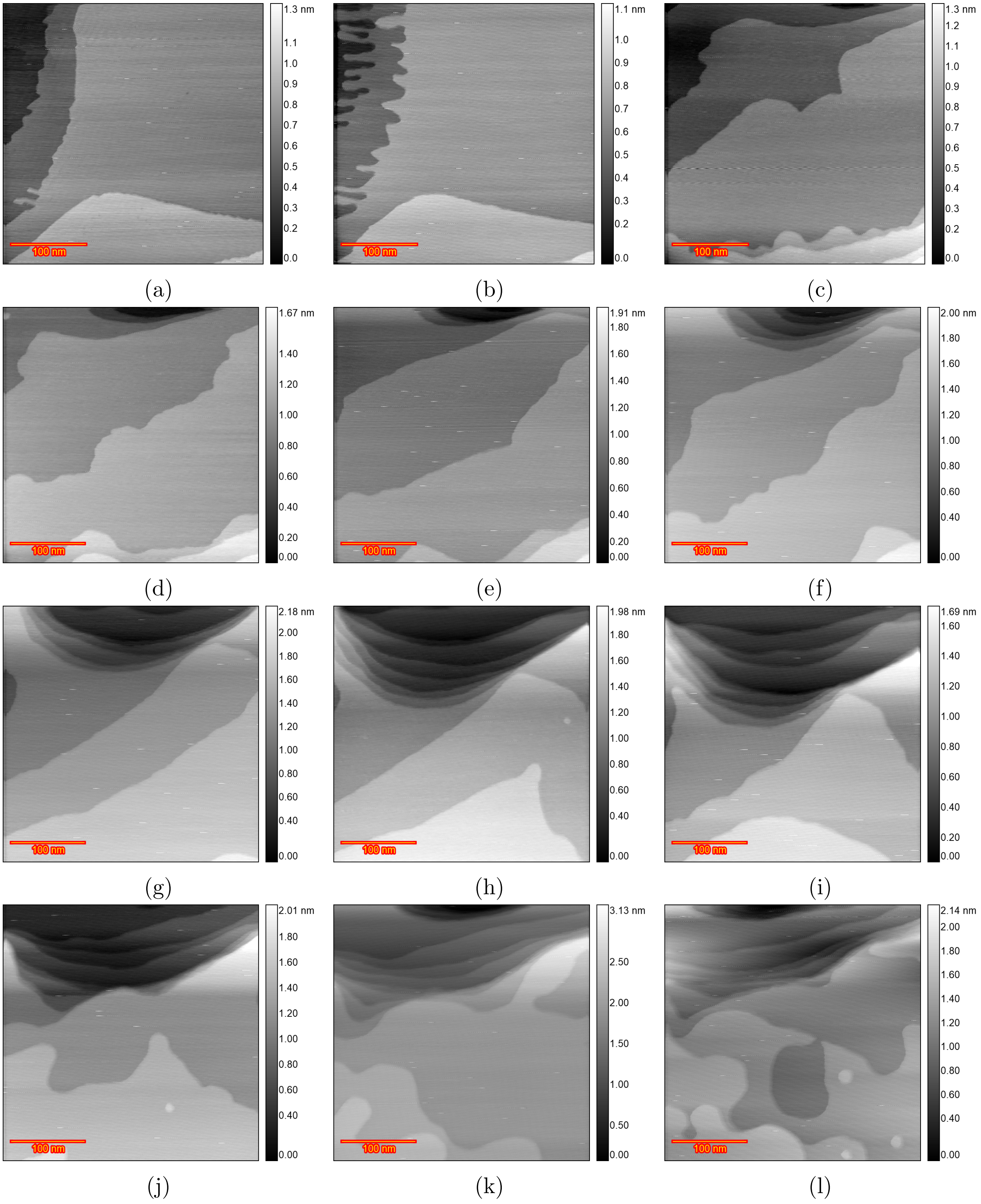

Figure S 13: Full sequence EC-STM images (350×350 nm) of experiment in 0.1 M $HClO_4$ containing 10 µM HCl. a) Sample surface just after annealing, b) lifted reconstruction at 0.9 V, c) after n ORCs from 0.8 to 1.65 V, n=10, d) n=20, e) n=35, f) n=50, g) n=75, h) n=100, i) n=125, j) n=150, k) n=175, l) n=200.

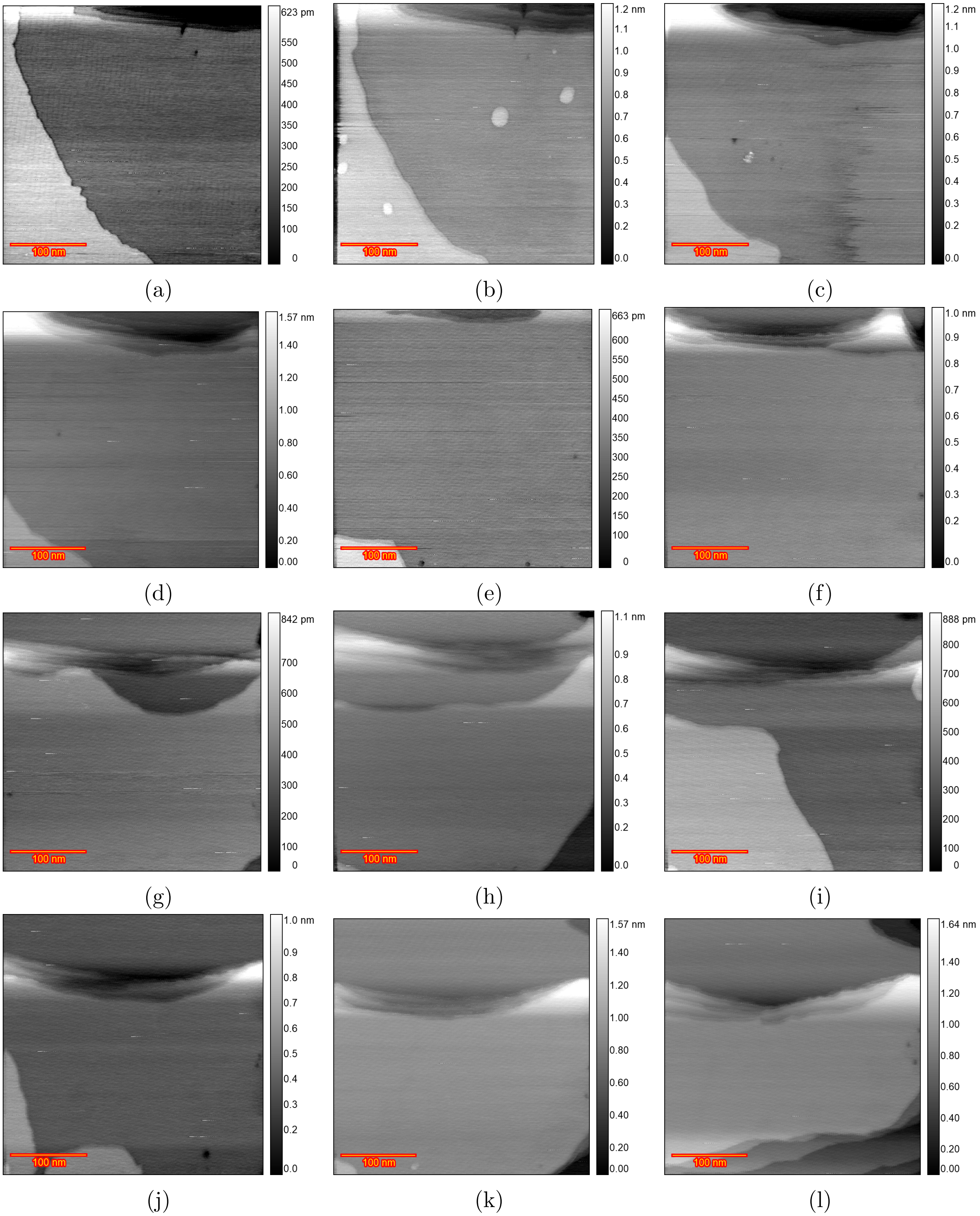

Figure S 14: Full sequence EC-STM images (350×350 nm) of experiment in 0.1 M $HClO_4$ containing 10 µM HCl. a) Sample surface just after annealing, b) lifted reconstruction at 0.9 V, c) after n ORCs from 0.8 to 1.65 V, n=10, d) n=20, e) n=35, f) n=50, g) n=75, h) n=100, i) n=125, j) n=150, k) n=175, l) n=200.

In a surprising discovery, repeating the experiment shown in Figure 8 revealed a behavior of growing vacancy islands similar to that observed in pure $HClO_4$ (experiments in Figures 4 and 5). At 0 V, terraces with a well-defined herringbone reconstruction divided by monatomic height step lines were evident (Figure S15a). At 0.9 V, some areas showed island formation due to the lifting of the reconstruction (Figure S15b). After performing 10 ORCs from 0.8 to 1.65 V at 50 mVs^{-1} and maintaining the potential at 0.8 V during imaging (Figure S15c), islands disappeared and the surface was partially covered with pits and some recession was observed in the step lines, similar to our previous observations in pure $HClO_4$ (Figures 4 and 5) which were affected by impurities. Imaging after 20 ORCs (Figure S15d) revealed the disappearance of pits and an increased recession rate of the step lines, consistent with what is expected from an $HClO_4$ solution containing Cl, as observed in Figure 8. This phenomenon is likely due to the increased mobility of gold surface atoms, influenced by the greater presence of locally adsorbed Cl^- on the terraces by the experiment time. After 75 ORCs Figure S15e shows that the step lines have receded at the rate observed in the experiment in Figure S 15 and the terraces appear pristine. However, after 125 ORCs (Figure S15f), some areas show pits, probably due to a lack of Cl^- ions on the newly exposed terraces following the etching of the front terrace through the receding step lines.

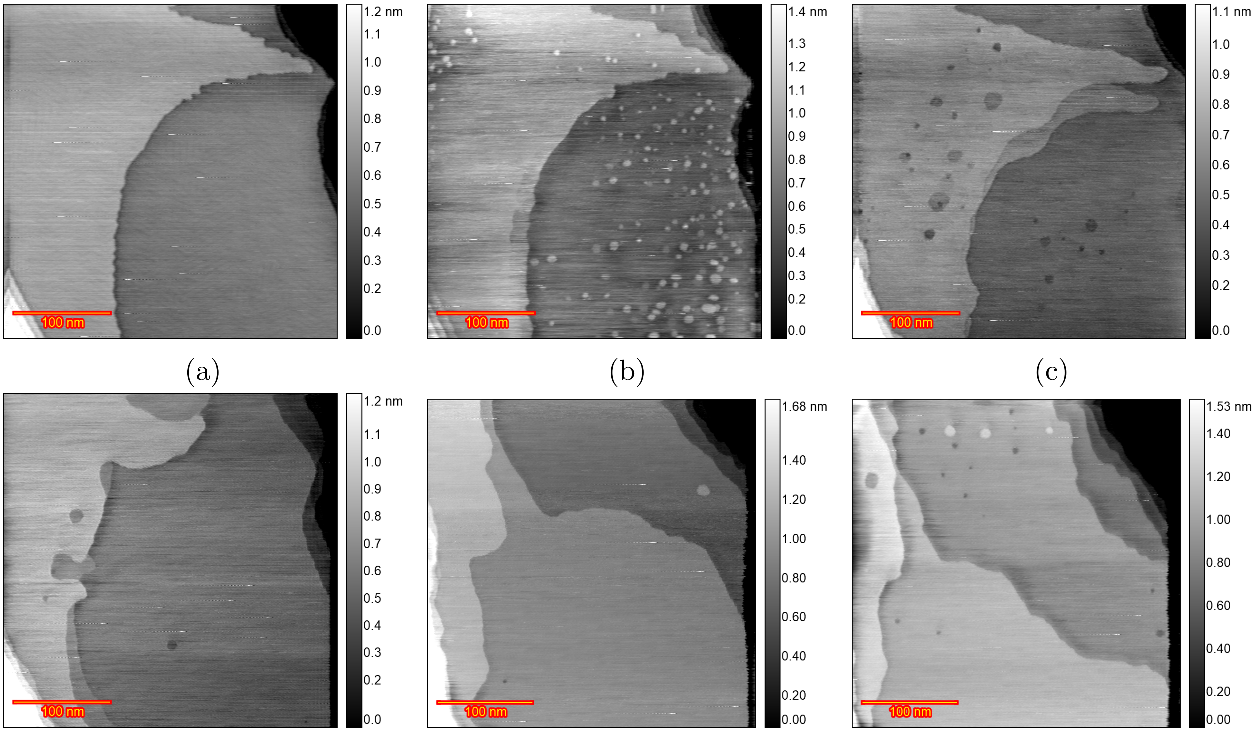

(a) (b) (c)

(d) (e) (f)

Figure S 15: EC-STM image(350×350 nm) of Au(111) in 0.1 M $HClO_4$ containing 10 µM HCl. a) Sample surface at 0.0 V vs RHE just after annealing. b) partially lifted reconstruction at 0.9 V. c) after n ORCs from 0.8 to 1.65 V and imaging at 0.8 V n=10 d) n=20 e) n=75 f) n=125.

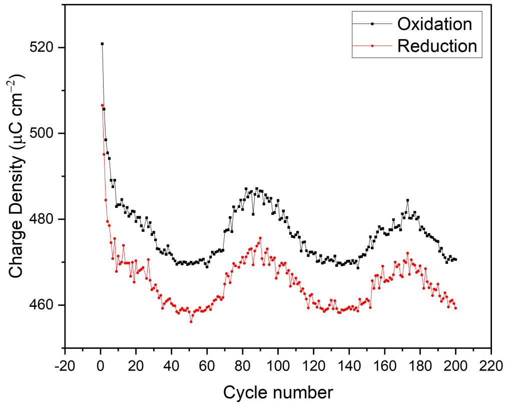

(a)

Figure S 16: Calculated oxidation and reduction charge densities of Au(111) in 0.1 M $HClO_4$ containing 10 µM HCl in the potential window of 0.9 to 1.7 V versus RHE for 200 ORCs.

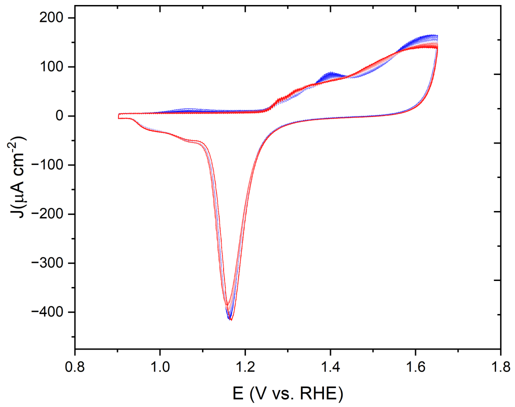

(a)

Figure S 17: Recorded CV of Au(111) in 0.1 M $HClO_4$ in the potential window of 0.9 to 1.65 V versus RHE during the EC-STM experiments in the EC-STM cell. The first CV presented in blue and the last one in red.

References

(1) Angerstein-Kozlowska, H.; Conway, B. E.; Hamelin, A.; Stoicoviciu, L. Elementary steps of electrochemical oxidation of single-crystal planes of Au—I. Chemical basis of processes involving geometry of anions and the electrode surfaces. *Electrochimica Acta* **1986**, *31*, 1051–1061.

(2) Angerstein-Kozlowska, H.; Conway, B. E.; Hamelin, A.; Stoicoviciu, L. Elementary steps of electrochemical oxidation of single-crystal planes of Au Part II. A chemical and structural basis of oxidation of the (111) plane. *Journal of Electroanalytical Chemistry and Interfacial Electrochemistry* **1987**, *228*, 429–453.
